# Studies on the Use of Flagellin as an Immunostimulant and Vaccine Adjuvant in Fish Aquaculture

**DOI:** 10.3389/fimmu.2018.03054

**Published:** 2019-01-09

**Authors:** Eakapol Wangkahart, Christopher J. Secombes, Tiehui Wang

**Affiliations:** ^1^Division of Fisheries, Department of Agricultural Technology, Faculty of Technology, Mahasarakham University, Mahasarakham, Thailand; ^2^Scottish Fish Immunology Research Centre, School of Biological Sciences, University of Aberdeen, Aberdeen, United Kingdom

**Keywords:** flagellin, immunostimulant, vaccine adjuvant, salmonids, cytokine, antimicrobial peptides, gene expression, inflammatory response

## Abstract

Immunostimulants and vaccines are important for controlling infectious diseases in fish aquaculture. In this study we assess the potential of flagellin to be used for such purposes in rainbow trout (*Oncorhynchus mykiss*). A recombinant flagellin from the salmonid pathogen *Yersinia ruckeri* (YRF) has been produced previously by us and shown to be a potent activator of inflammatory cytokines, acute phase proteins and antimicrobial peptides *in vitro*. Here we show that YRF is the most potent inflammatory activator of three bacterial PAMPs (LPS, peptidoglycan and flagellin) tested. The host response to flagellin was next studied *in vivo*. The YRF modulated gene expression was examined in two systemic (spleen and liver) and two mucosa-associated (gills and skin) tissues. YRF injection initiated a transient systemic inflammatory response with key pro-inflammatory cytokines (IL-1β, TNFα, IL-6, and IL-11 etc.) and chemokines (CXCL_F4 and CXCL-8) induced rapidly (by 6 h) but subsiding quickly (by 24 h) in multiple tissues. Consequently, a variety of anti-microbial pathways were activated systemically with heightened expression of acute phase proteins, antimicrobial peptides and complement genes in multiple tissues, which was sustained to 24 h in the liver and mucosal tissues. The Th17 cytokine IL-17A/F1 was also induced in the spleen and liver, and Th2 cytokine IL-4/13 was induced in the liver. However, the anti-inflammatory IL-10 and the Th1 cytokine IFNγ were refractory. A secreted form of TLR5 (TLR5s) was induced by flagellin in all tissues examined whilst the membrane form was refractory, suggesting that TLR5s may function as a negative feedback regulator. Trout liver appeared to be an important organ responding to flagellin stimulation, with marked induction of IL-11, IL-23P19, IL-17C1, SAA, and cathelicidin-2. YRF induced a strong antibody response. These antibodies reacted against the middle domain of YRF and were able to decrease YRF bioactivity. Intact YRF was necessary for its bioactivity, as deletion of the N-terminal, C terminal or middle domain of YRF led to functional loss. This study suggests that flagellin could be a potent immunostimulant and vaccine adjuvant for fish aquaculture.

## Introduction

Aquaculture accounts for more than half of the fish consumed worldwide and contributes greatly to the supply of affordable protein in developing countries (1). The farmed fish are kept at high population densities that increase the risk of disease outbreaks caused by infectious bacteria, viruses, parasites and fungi (2–4), hence infectious disease is one of the major limiting factors in aquaculture. Multiple measures must be taken to ensure the health of these fish and the use of immune-stimulants and vaccination represent two important strategies for controlling diseases in aquaculture (2, 3, 5–13).

Fish have a strong innate immune system that can cope with a large variety of infectious agents. However, many pathogens have developed evasion mechanisms to resist innate immune defenses, and in such cases the adaptive immune system, that evolved for the first time in early vertebrates, must come into play to fight these pathogens (8). The innate and adaptive immune systems are cross-linked, and the magnitude and specificity of the signals perceived by the innate immune cells following infection or vaccination shape subsequent adaptive immune responses (9).

Immune-stimulants are chemical compounds that activate leukocytes and hence may render animals more resistant to infections. The innate immune response is initiated upon recognition of pathogen-associated molecular patterns (PAMPs), such as double stranded RNA, flagellin, lipopolysaccharide (LPS) and β-glucans (9, 14), by pattern recognition receptors (PRRs) such as Toll-like receptors (TLR), C-type lectin receptors (CLRs), NOD-like receptors (NLR) and RIG-I-like receptors (RLR) (15). In the presence of PAMPs, the immune system will respond as if challenged by a pathogenic microbe. Hence, PAMPs, when administrated prior to an infection, may elevate defenses and function as immune stimulants. They can also function as adjuvants when formulated with vaccines, to elevate the specific adaptive immune response (11–13).

Bacterial flagellin is the major structural protein in the flagellum of Gram positive and negative bacteria (8, 16). Due to its presence in diverse bacterial species and high abundance in bacterial cells, flagellin is a potent PAMP and a major target of the host immune system. Monomeric flagellin (30–60 kDa, dependent upon the taxa of the bacterium) contains in general an N-terminal D0/D1, a middle D2/D3 and a C-terminal D1/D0 domain. The last ~40 amino acids of each terminus of the flagellin molecule form the D0 domain. The D1 domain constitutes the next ~100 residues from the N-terminus and 50 residues from the C-terminus. The D0 and D1 domains are key for assembly of the helical filamentous structure and hence are highly conserved across different species of bacteria, and contain primarily α-helical structures. In contrast the D2 and D3 domains have high sequence diversity and can be absent in some bacterial species, such as *B. cereus* (17–20). The hypervariable D2 and D3 domains are composed of β-sheets bearing adhesion-like properties and are essential for flagellin immunogenicity (16, 17).

In mammals, extracellular flagellin is recognized by TLR5 expressed by antigen-presenting cells and T cells (21, 22). Mammalian TLR5 is a plasma membrane-localized PRR (TLR5M) that possesses an extracellular domain with leucine-rich repeats (LRRs), a transmembrane region, and a cytoplasmic signaling domain termed the Toll/interleukin-1 receptor homology (TIR) domain. Crystal structure analysis showed that two flagellin molecules simultaneously bind two TLR5 receptors on the D1 domain, forming a 2:2 complex. In addition to the D1 domain, deletion mutant experiments revealed that the D0 domain is also required to produce maximum TLR5-mediated signaling (17–19). The activation of TLR5 mediates the production and secretion of pro-inflammatory cytokines, chemokines and other mediators for the development of an effective immune response (21, 23). In rainbow trout (*Oncorhynchus mykiss*) and other teleost species, two TLR5 genes are present in the genome (24–29). One (TLR5M) encodes for an extracellular LRR domain, a transmembrane region, and a cytoplasmic TIR domain as seen in mammalian TLR5. The second encodes only the LRR in the extracellular domain and hence is a soluble form of TLR5 (TLR5S). Trout TLR5M is widely expressed in all tissues whereas TLR5S is mainly expressed in liver (24, 25). Both the TLR5M and TLR5S recognize flagellin from *Vibrio anguillarum* a Gram-negative bacterium (24).

The evaluation of flagellin as a vaccine candidate, and as a vaccine adjuvant have been examined in fish recently (16, 30–35). Flagellin has also been shown to induce non-specific protection to a variety of bacterial pathogens *in vivo* in rainbow trout (36). However, the immune pathways elicited and the mechanisms responsible are largely unknown, with only few pro-inflammatory genes and tissues studied (14, 15, 37).

A recombinant flagellin from the fish pathogen *Yersinia ruckeri* (YRF) was produced in our lab and shown *in vitro* to upregulate the transcript level of a large number of pro-inflammatory cytokines, APPs, AMPs and members of the IL-12 cytokine family in the monocyte/macrophage-like cell line, RTS-11 (23). In the present study the immunomodulatory effects of flagellin were explored further *in vivo* in several major immune tissues, namely spleen, liver, gills and skin. In teleost fish the kidney, spleen and liver are major systemic lymphoid tissues containing many immune cell types that are vital components for initiating immune reactions within the immune system (38, 39). Mucosa-associated lymphoid tissues (gut, gills, nares and skin) are also important to prevent invasion of pathogens from the surrounding environment of the host (40–42). Hence two systemic and two mucosa-associated tissues were chosen for study of the immune-modulatory effects following YRF injection *in vivo*. Our results show that YRF activates a systemic inflammatory response and multiple antimicrobial pathways *in vivo*. To pave the way for the use of YRF as an immune stimulant or a vaccine adjuvant, we also investigated the antigenicity of full-length YRF and the bioactivity of YRF deletion mutants. The results showed that fish could produce high serum antibody titres against flagellin after vaccination, and that these antibodies recognize the middle D2/D3 domain of the flagellin and can decrease flagellin bioactivity. However, deletion of either the N-terminal, C terminal or the middle domain of YRF led to loss of the proinflammatory activity. The impact of these results on the potential use of flagellin as an immune-stimulant or vaccine adjuvants in fish aquaculture is discussed.

## Materials and Methods

### Experimental Fish

Healthy rainbow trout were bought from the Mill of Elrich Trout Fishery (Aberdeenshire, Scotland, United Kingdom) and kept in 1-m-diameter fiberglass tanks with recirculating freshwater at 14°C at the Scottish Fish Immunology Research Centre, University of Aberdeen, UK. Fish were fed twice daily with a commercial diet (EWOS) and were given at least 2 weeks to acclimate before use. Routine screening of head kidney swabs showed no bacterial presence (43).

### Production of Recombinant *Y. ruckeri* Flagellin and Its Mutants

The construct pTri-YRF for expression of full-length recombinant *Y. ruckeri* flagellin (YRF) and the production of YRF was described previously (23). The mutant YRF constructs, YRF-N, YRF-C and YRF-NC, were prepared from pTRI-YRF by PCR using the Q5 high fidelity enzyme (New England Biolabs, United Kingdom) and re-ligation, using primer pairs GCCAGTTCCGCTCATCACCAC/GGAACGGAAGTTACCGTTAACCATC (YRF-N), GCCCATGGTATATCTCCTTTGATTGT/GATAACCGCACGGCAGCCA (YRF-C), and CAAGACTTTAATGCCGTTGAAATCGGT/GTTGAAGCCAAAGGTTTTGACGTATTGA (YRF-NC), respectively. Whilst the YRF-N and YRF-C have the C-terminal and N-terminal D0/D1 domains deleted, respectively, the YRF-NC has the middle D2/D3 removed and replaced with a GS linker [SGGGGSGGGGSGGGGS, (44)]. All the muteins have a his-tag (ASSAHHHHHHHHHH) at the C-terminus for purification. A multiple alignment of YRF and its muteins is provided in Figure S1. Following sequence confirmation, the transformation of BL21 Star (DE3) competent cells (Invitrogen), induction of recombinant protein production, purification under denaturing conditions, refolding, re-purification under native conditions, SDS-PAGE analysis of proteins and quantification of protein concentration were as described previously (23).

### Stimulation of RTS-11 Cells

The monocyte/macrophage-like cell line, RTS-11, from rainbow trout spleen was cultured in Leibovitz (L-15) medium (Invitrogen, United Kingdom) plus 30% fetal calf serum (FCS; Labtech International, United Kingdom) and antibiotics (100 U/ml penicillin and 100 μg/ml streptomycin; Invitrogen, UK) at 20°C, and passaged as described previously (45). For experiments, cells were collected by centrifugation (200 × g, 5 min), re-suspended in L-15 containing 10% FCS to 1 x 10^6^ cells/ml, and seeded into 12-well cell culture plates at 2 ml/well. Overnight cell cultures were stimulated with 100 ng/ml YRF, 5 μg/ml ultrapure lipopolysaccharide (LPS, Invivogen) from *E. coli* 0111:B4, and 5 μg/ml peptidoglycan (PGN, Invivogen) purified from the Gram-positive bacterium *Staphylococcus aureus*, for 1, 2, 4, 8, and 24 h. Subsequently the cells were stimulated with different concentrations of YRF and its muteins for 4 h. The stimulation was terminated by dissolving the cells in TRI reagent (Sigma, United Kingdom). The RNA preparation and cDNA synthesis was as described previously (23, 46).

### Administration of YRF *in vivo* and Sampling

Twenty eight rainbow trout (~210 g) were randomly divided into two groups. Fish were injected intraperitoneally (ip) with 200 μl of phosphate-buffered saline (PBS, pH 7.4) or PBS containing 10 μg of YRF per fish. The dose chosen was based on the finding that 50 ng/ml *in vitro* induces the highest gene expression changes in most of the genes examined (23), and equates to 50 ng/g body weight *in vivo*. Whilst this dose is at the low end of those used previously in salmon and trout (10–50 μg per fish) (16, 36), the results demonstrate it was highly effective. Seven fish per group were killed at 6 and 24 h post injection, and the gills, skin, spleen and liver were taken and homogenized in TRI reagent with 5 mm stainless steel beads using a TissueLyser II (Qiagen) as described previously (46).

### Real-Time PCR Analysis of Gene Expression

Total RNA extraction, cDNA synthesis and real-time PCR analysis of gene expression were as described previously (47, 48). The expression of cytokines, antimicrobial peptides (AMPs), acute phase proteins (APPs), complement components, arginase genes, and suppressor of cytokine signaling (SOCS) genes as well as the house keeping gene elongation factor-1α (EF-1α), was examined. The primers for real-time PCR are given in Table S1, with at least one primer per pair designed to cross an intron to ensure genomic DNA could not be amplified under the PCR conditions used. The expression of each gene was initially normalized to that of EF-1α, and then shown as a fold change by calculating the average expression level of the treated samples divided by that of time-matched controls.

### Immunization and Antiserum Preparation

Ten rainbow trout (~200 g) were ip injected with 90 μg/fish of YRF mixed with 0.2 ml of complete Freund's adjuvant (CFA, Sigma, United Kingdom) (YRF+CFA). A control group of 10 fish was ip. injected with CFA alone. The fish were bled from the caudal vein 3 months post immunization. Blood samples were incubated at 4°C overnight and then centrifuged at 5,000 rpm for 10 min at 4°C. Serum was collected and stored at −80°C until use.

### Enzyme-Linked Immunosorbent Assay (ELISA)

YRF-specific IgM antibody titres in serum samples were determined by ELISA using mouse anti-trout IgM (clone I-14) (49). Briefly, high binding capacity 96 well-plates (Thermo scientific) were coated with 1,000 ng/well of YRF in coating buffer (pH 9.0, 100 mM NaHCO_3_, 12 mM Na_2_CO_3_) for 2 h at 37°C and washed 3 times with wash buffer (PBS containing 1% (w/v) skimmed milk and 0.05% (v/v) Tween 20) and then blocked for 2 h at 37°C with 5% (w/v) skimmed milk in wash buffer. Plates were washed 3 times with wash buffer before addition of serum samples. Individual fish serum was serially diluted 2-fold in 1% milk in 1X PBS. Each dilution was added to YRF coated plates (50 μl/well) in duplicate and incubated at 4°C overnight. Plates were then washed 3 times with wash buffer and mouse anti-trout IgM added (50 μl/well) and incubated for 2 h at 37°C. After washing 3 times with wash buffer, the detection antibody (goat anti-mouse IgG labeled with horseradish peroxidase, Sigma, UK) diluted 1:3,000 in 1X PBS, 1% milk was added (50 μl/well) and incubated for 1 h at 37°C. The plates were again washed 3 times with wash buffer and developed by adding 50 μl/well of 3, 3′, 5,5′-Tetramethylbenzidine (TMB) Liquid Substrate, Supersensitive, for ELISA (Sigma, UK) to each well for 30 min. The reaction was stopped by adding 50 μl/well of 0.5 M sulphuric acid and the color reaction read at 450 nm in an ELISA plate reader (SoftMax Pr0 5.3). The antibody titres were determined as the reciprocal of the maximal serum dilution that exceeded double the reading of the negative control.

### Neutralization Assay

The potential of antisera (see section Immunization and Antiserum Preparation) to neutralize YRF bioactivity was test in RTS-11 cells. Ten nanogram of YRF was mixed with 100 μl anti-YRF sera or control sera, or L-15 cell culture medium and incubated at room temperature for 1 h. The samples were then added (in quadruplicate) to each well of a 12-well plate containing RTS-11 cells prepared as above (section Stimulation of RTS-11 Cells). A matched serum or L-15 sample without YRF was also added to RTS-11 cells to function as additional controls. The incubation was terminated at 4 h by dissolving the cells in TRI reagent® (Sigma, United Kingdom). The expression of IL-1β1 and IL-1β2 was determined by RT-qPCR as described above.

### Western Blot

The binding capacity of antisera from rainbow trout immunized with YRF/CFA to YRF and its muteins was examined by Western blot analysis. The preparation of full-length YRF and its deletion mutants YRF-N, YRF-C and YRF-NC was as described above (section Production of Recombinant *Y. ruckeri* Flagellin and Its Mutants). Four concentrations (1,000, 500, 250, and 125 ng) of YRF and mutated proteins (~1,000 ng) were separated by SDS-PAGE. Another un-related recombinant flagellin (Flagellin-B) prepared in a similar way as YRF was also included as a control. Briefly, each sample was mixed with NuPAGE LDS Sample Buffer (Invitrogen, United Kingdom) and 0.5% of 2-ME, boiled at 95°C for 15 min, and loaded into the wells of a NuPAGE™ Novex™ 4–12% Bis-Tris Protein Gel (Invitrogen, United Kingdom), along with SeeBlue® Plus2 Pre-stained Protein marker (Invitrogen, United Kingdom). The gel was run in 1X NuPAGE® MES SDS Running Buffer, at 200 Volts for 30 min. The protein gel was either stained with Imperial protein stain (Thermo Scientific, UK), or transferred to Hybond®-P polyvinylidene difluoride (PVDF) membranes (Ambion) using a NuPAGE® Transfer Buffer system (Invitrogen) as recommended by the manufacturer.

The PVDF membrane was blocked with 5% skimmed milk in PBS (pH 7.2) containing 0.05% Tween 20 (PBST) for 1 h and washed 3 times with 1X PBST. The membrane was then incubated with the trout anti-YRF sera (diluted 1:1,000 in PBST) overnight at 4°C. After subsequent addition of mouse anti-trout IgM (I-14 diluted 1:1,000 in PBST) and goat anti-mouse IgG labeled with horseradish peroxidase (diluted 1:2,000, Sigma, UK), with three washes between treatments, the membrane was washed 5 times with 1X PBST and antibody binding detected with a SuperSignal™ West Pico Chemiluminescent Substrate kit (Thermo Scientific, United Kingdom).

### Statistical Analysis

The data were statistically analyzed using the SPSS Statistics package 24 (SPSS Inc., Chicago, Illinois). The analysis of real-time PCR data was as described previously (47, 48). To improve the normality of data, real-time quantitative PCR measurements were scaled, with the lowest expression level in a data set defined as 1, and log2 transformed. One way-analysis of variance (ANOVA) and the Bonferroni *post-hoc* test were used to analyse the gene expression data, with *P* < 0.05 between treatment and control groups considered significant. Genes significantly modulated by YRF *in vivo* were selected and their fold changes were log2 transformed and subjected to hierarchical clustering analysis using the Morpheus program at https://software.broadinstitute.org/morpheus.

## Results

### *Yersinia ruckeri* Flagellin (YRF) Is the Most Potent Bacterial Derived PAMP to Activate Pro-Inflammatory Genes in RTS-11 Cells

**Figure 1:**
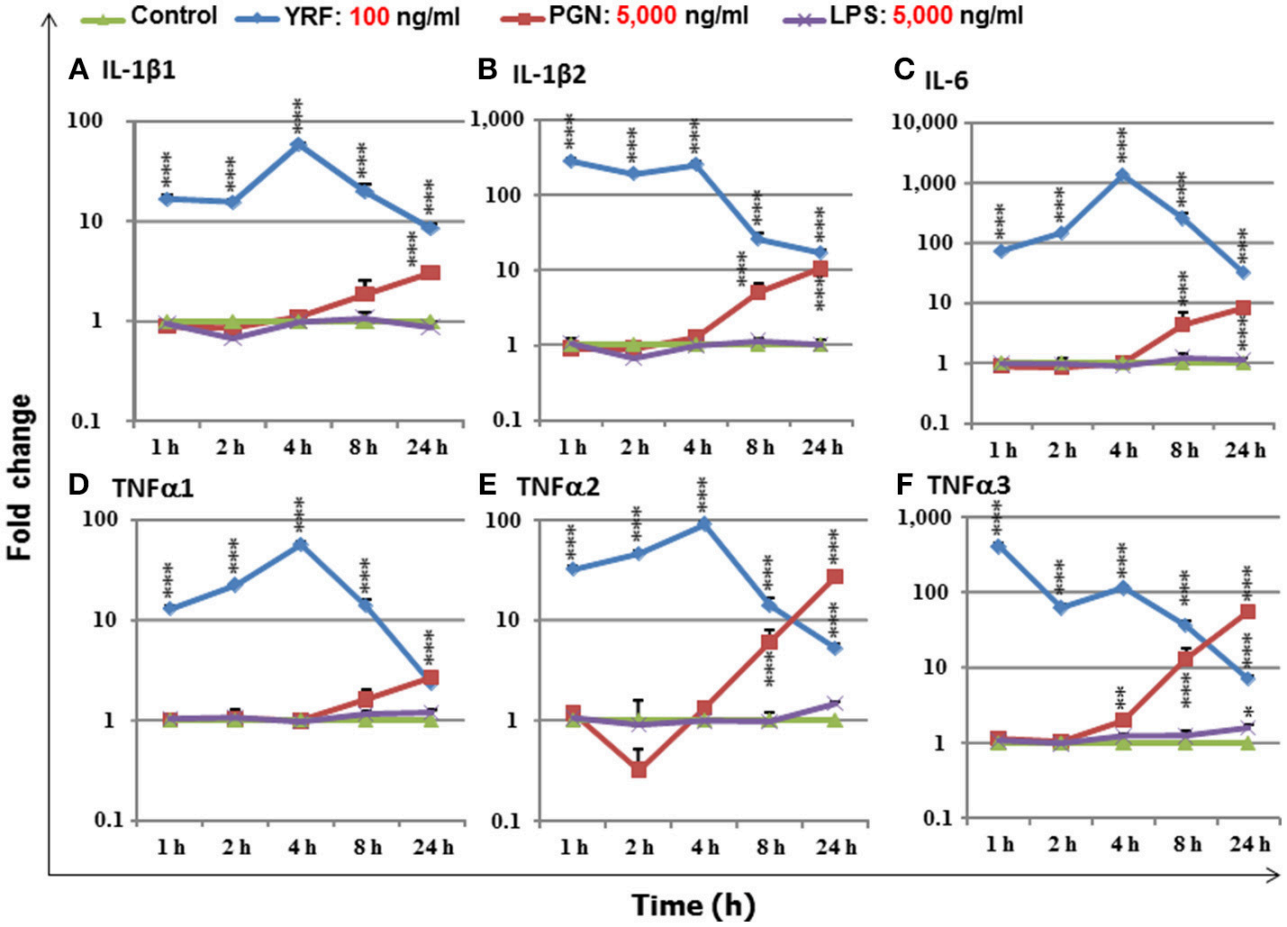
The expression of IL-1β1 **(A)**, IL-1β2 **(B)**, IL-6 **(C)**, TNFα1 **(D)**, TNFα2 **(E)**, and TNFα3 **(F)**, in response to stimulation by YRF, LPS and PGN in RTS-11 cells. RTS-11 cells were stimulated with 100 ng/ml of YRF, 5,000 ng/ml of ultrapure LPS or 5,000 ng/ml of PGN for 1, 2, 4, 8, and 24 h. The expression levels were quantified by RT-qPCR and expressed as a fold change that was calculated as the average expression level of stimulated samples divided by that of the time-matched controls. The results are presented as the average + SEM from four wells of cells. Differences between stimulated samples and controls were tested by One way-ANOVA followed by the Bonferroni *post hoc* test. ^*^p < 0.05, ^**^p < 0.01, and ^***^p < 0.001.

LPS, PGN and flagellins are bacterial derived PAMPs that can activate an inflammatory response in mammals and have the potential to be used as an immune stimulant or vaccine adjuvants in fish aquaculture. To compare the immune stimulatory potency of these PAMPs, a fish macrophage-like cell line (RTS-11) was incubated with an optimal dose (100 ng/ml, 23) of YRF, or 50-fold higher dose (5,000 ng/ml) of ultrapure LPS and PGN in a time course from 1 to 24 h. The expression of major proinflammatory cytokines, IL-1β1-2 (50), TNFα1-3 (51), and IL-6 (52) was examined. YRF rapidly activated all the proinflammatory cytokines from 1 h-24 h, with a peak already seen at 1 h for IL-1β2 and TNFα3, or at 4 h for other genes (Figure 1) as seen previously (23). PGN also activated all the inflammatory cytokines examined but at later time points, 4–24 h for TNFα3, 8–24 h for IL-1β2, IL-6 and TNFα2, and 24 h for IL-1β1 and TNFα1, and with less potency, eg IL-6 expression was increased over 1,000-fold by YRF but only 10-fold by PGN (Figure 1). Ultrapure LPS only slightly increased the expression of TNFα3 at 24 h post stimulation and had no effect on the other cytokines examined. The expression of proinflammatory cytokines in samples stimulated with YRF at each time point was higher than that with LPS from 1 to 24 h, and with PGN from 1 to 8 h. At 24 h, YRF stimulated samples expressed higher levels of IL-1β1, IL-1β2 and IL-6, similar level of TNFα1, but lower levels of TNFα2 and TNFα3, compared to PGN stimulated samples. These expression profiles suggest that flagellin (YRF) is the most potent PAMP tested *in vitro* to date in rainbow trout.

### Systematic Activation of Early Pro-Inflammatory Pathways *in vivo* by YRF

After injection with YRF the fish behaved normally and no side-effects were observed postmortem. The expression profiles of 120 trout genes were determined by RT-qPCR in spleen, liver, gills and skin, at 6 h and 24 h post injection (hpi). The genes analyzed included the known cytokines and SOCS genes in trout, and genes of the acute phase response and antimicrobial defense. The time points chosen were based on our previous study that showed YRF activates a rapid increase in proinflammatory genes *in vitro* (23). Thus the early response (at 6 hpi) and late response (at 24 hpi) were studied here. Detailed fold changes for each genes analyzed are provided in Table S2.

#### Expression of the Main Proinflammatory Cytokines

**Figure 2:**
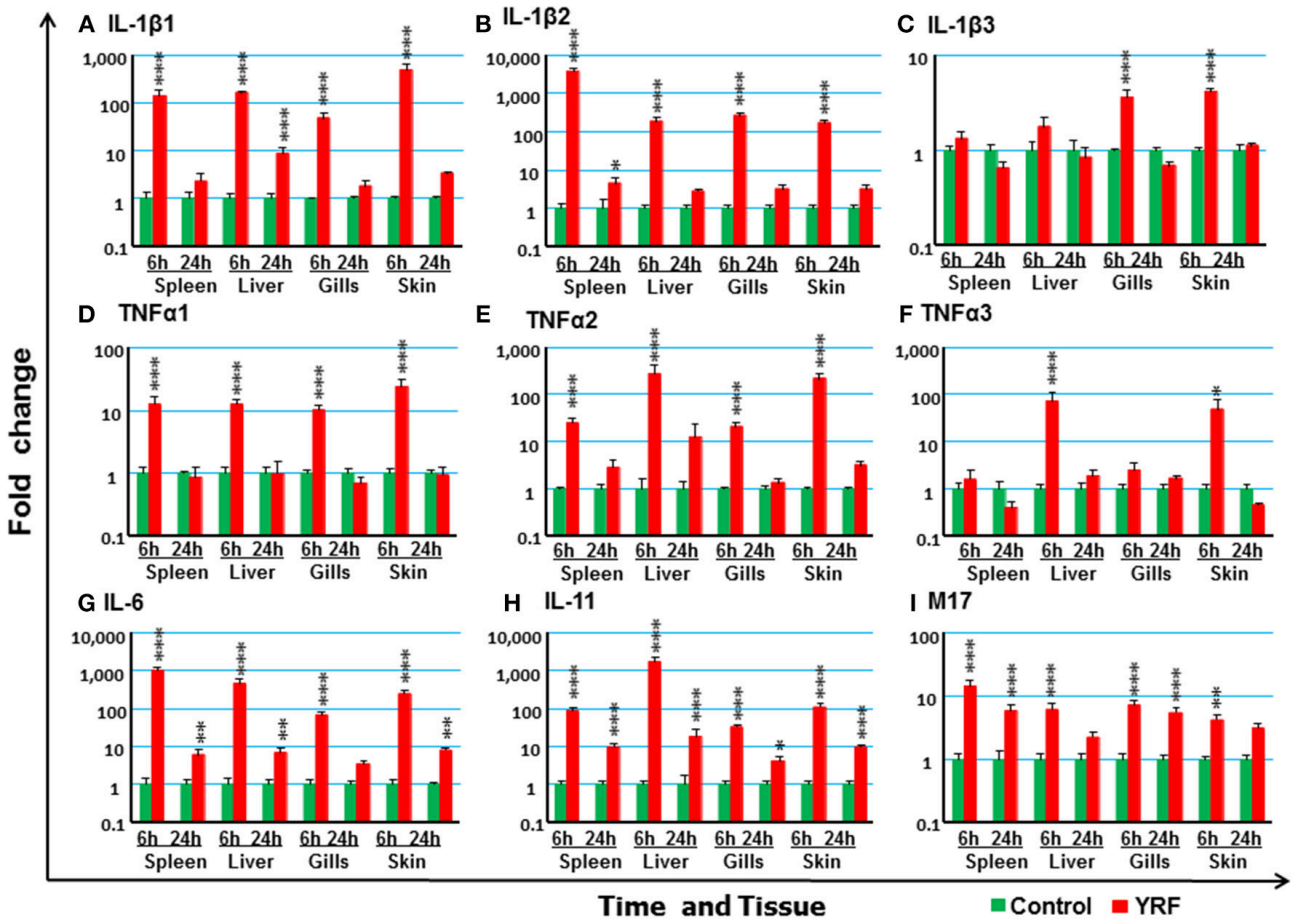
The activation of major proinflammatory cytokines *in vivo* by YRF. Rainbow trout were ip injected with 10 μg YRF/fish in PBS or PBS only as control. The fish were killed, and spleen, liver, gills and skin samples were taken from each fish at 6 and 24 h post injection. The expression of IL-1β1 **(A)**, IL-1β2 **(B)**, IL-1β3 **(C)**, TNFα1 **(D)**, TNFα2 **(E)**, TNFα3 **(F)**, IL-6 **(G)**, IL-11 **(H)**, and M17 **(I)** in each tissue was examined by RT-qPCR. A fold change was calculated as the average expression level of YRF injected fish divided by that of time matched controls. The means + SEM of six fish are shown. Significant differences between injected fish and controls were tested by one way-ANOVA followed by the Bonferroni *post hoc* test and are shown over the bars as; ^*^p < 0.05, ^**^p < 0.01, and ^***^p < 0.001.

The activation of TLR5 by flagellin triggers an inflammatory response and activates the expression of fish immune relevant genes *in vitro* (23). Therefore, following injection of YRF the expression of major proinflammatory cytokines of the IL-1 family, IL-6 family and TNFα family was first examined. Five IL-1 family members, three IL-1β paralogues (50), IL-18 (53) and a novel IL-1 family member (nIL-1Fm) that antagonizes IL-1β activity (54) are present in rainbow trout. The expression of IL-1β1 and IL-1β2 was rapidly increased at 6 hpi in all tissues. This activation subsided to control levels at 24 hpi with the exception of IL-1β1 in the liver and IL-1β2 in the spleen where the expression remained higher than the control (Figures 2A,B). The expression of IL-1β3 was also rapidly induced but in a tissue specific way at 6 h. Thus IL-1β3 expression was only induced at 6 h in the gills and skin (Figure 2C). IL-18 expression was induced only in spleen at 6 h and in the liver at 24 h (Table 1).

**Table 1:** Fold change of transcript expression of selected genes in the spleen, liver, gills, and skin of fish injected ip with YRF.

**Tissue**	**Spleen**		**Liver**		**Gills**		**Skin**	
**Time**	**6 h**	**24 h**	**6 h**	**24 h**	**6 h**	**24 h**	**6 h**	**24 h**
IL-18	2.7^***^	1.2	1.5	4.9^***^	1.2	1.2	1.2	1.5
CNTF	0.5	0.9	0.8	0.6	1.0	0.6	1.0	0.6
P35B1	2.4	0.3	9.0	0.1	1.6	0.8	13.9	0.9
P28A	2.3	0.9	ND	ND	13.4	1.5	7.5	0.4
P28B	5.9	2.0	ND	ND	ND	ND	ND	ND
CXCL_F1a	22.2	103.0	1.5	1.0	0.6	0.7	1.6	0.8
CXCL_F1b	0.8	12.2	0.7	2.7	1.5	2.6	1.2	1.6
CXCL_F2	1.1	0.3	2.0	0.7	1.1	0.5	0.7	0.6
CXCL12a	0.9	0.4	1.8	0.4	0.8	1.6	0.9	1.5
CXCL12b	0.8	0.4	0.7	1.2	0.8	1.7	0.5	0.8
CXCL14	1.6	0.7	0.8	0.6	1.0	1.5	0.9	1.8
IFNγ1	1.3	0.7	1.0	3.8	0.3	0.9	0.2	0.4
IFNγ2	1.0	1.9	1.3	1.3	0.4	0.8	0.5	0.5
IL-17A/F1b	ND	17.5	ND	ND	1.2	0.6	0.3	0.6
IL-17A/F2a	0.6	0.7	1.9	11.4^***^	0.4	0.5	0.2	0.4
IL-17A/F2b	0.8	1.1	2.0	2.2	0.9	1.0	0.5	0.9
IL-17A/F3	0.6	1.2	1.6	0.6	0.7	1.1	0.4	0.7
IL-17N	1.9	1.6	1.1	1.2	2.2	1.0	0.9	0.8
IL-17D	0.9	0.4	0.4	0.6	1.0	0.8	0.6	0.4
IL-2	1.1	0.7	1.2	1.1	1.0	0.6	1.0	1.1
IL-15	1.0	0.6	2.7^***^	8.4^***^	2.4^*^	0.7	1.2	1.0
IL-20	6.5	1.2	2.0	2.1	1.0	1.8	1.0	1.1
IL-21	1.6	0.9	1.7	1.4	1.5	0.8	0.9	0.9
IL-34	2.9^***^	1.0	18.7^***^	0.7	1.7	1.1	3.4^***^	1.5
CISHb1	1.9	2.5	1.4	0.8	1.2	0.6	1.2	0.8
CISHb2	2.8	2.1	0.9	1.0	1.4	1.0	1.8	1.0
SOCS1B	1.2	1.4	0.5	2.8	0.8	1.2	0.9	1.3
SOCS2A1	2.4	3.7	1.1	0.3	1.3	1.8	1.0	1.3
SOCS2A2	0.6	0.5	3.3	0.2	1.4	1.2	0.7	0.8
SOCS2B	1.0	0.2	2.5	0.4	1.2	0.3	1.1	0.3
SOCS3B1	1.5	1.5	1.7	3.8	1.0	2.5	2.2	3.4
SOCS4	1.3	0.3	0.3	0.5	1.8	1.7	0.8	1.3
SOCS5A1	1.2	1.5	0.1	2.3	1.2	1.8	0.8	2.4
SOCS5A2	0.6	1.3	0.1	0.9	0.9	1.6	1.0	1.6
SOCS5B1	1.0	1.0	0.4	1.1	1.0	1.4	1.4	1.3
SOCS5B2	1.4	1.1	0.7	1.4	2.8	1.7	2.8	2.4
SOCS6B1	1.0	1.0	0.4	0.8	1.2	1.4	0.5	0.9
SOCS7A1	1.4	1.0	0.6	1.2	1.7	1.3	1.6	1.3
SOCS7A2	0.9	0.6	1.5	0.9	1.6	1.0	0.9	1.2
SOCS7B1	3.0	2.0	1.5	1.6	1.8	2.5	0.7	1.4
SAP2	1.8	1.8	0.9	1.7	1.1	0.9	0.9	2.1
β-defensin 1	ND	0.0	ND	ND	1.8	0.3	0.9	1.2
β-defensin 2	3.1	5.4	1.5	0.8	0.8	0.7	0.3	1.2
β-defensin 3	4.4	1.3	0.9	1.8	7.6	3.3	3.0	1.3
LEAP2A	0.6	1.3	2.6	0.04^***^	0.5	0.8	1.3	1.1
LEAP2B	0.8	0.2	1.2	0.05^***^	1.3	0.6	0.7	0.6
Bf-2	1.9	1.9	1.7	1.4	1.4	0.9	0.2	0.5
C1 inhibitor	2.8	1.1	1.0	0.7	0.8	1.2	0.8	1.3
C4	0.8	0.9	1.2	0.9	1.2	1.9	0.6	3.2
C5	7.8	1.4	1.3	1.3	1.1	0.7	0.2	1.2
C7-2	1.2	0.7	0.9	0.8	0.9	1.2	1.3	0.7
C8α	5.4	1.5	0.9	0.8	1.3	0.3	0.6	0.7
C8β	1.9	1.0	1.3	0.6	1.2	0.9	0.7	0.8
C8γ	0.9	0.5	1.2	0.6	1.2	1.4	1.1	0.8
C9	1.0	1.0	1.5	1.8	0.6	0.9	0.6	0.5
C3-3	4.0	3.7^*^	1.0	1.3	0.6	2.0	0.4	1.4
C3-4	6.2^*^	2.0	1.1	0.5	1.8	0.9	0.9	0.6
C3aR	1.8	1.7	1.5	1.5	2.5	1.1	1.9	1.6

*The fold change was calculated as the average expression level of YRF injected fish divided by that of time-matched controls (N = 6). A number in bold with asterisks indicates significant differences between YRF injected fish and controls as follows*;

* *p < 0.05*,

** p < 0.01, and

*** *p < 0.001 (One-way-ANOVA with Bonferroni correction)*.

*ND, not determined due to low expression levels*.

ND^*^, detectable in YRF injected fish but not detectable in time-matched controls.

The expression of TNFα paralogues was also rapidly activated. The expression profiles of both TNFα1 and TNFα2 were similar and both were induced at 6 h in all four tissues examined (Figures 2D,E). However, TNFα3 expression was only induced at 6 hpi in the liver and skin (Figure 2F). The expression had returned to control levels by 24 hpi for all genes.

IL-6 expression was rapidly and highly induced at 6 hpi in the spleen, liver, gills and skin of injected fish (Figure 2G). The heightened expression was retained in the spleen, liver and skin to 24 hpi, albeit at lower levels. Similarly, the expression level of IL-11 (55) was also significantly up-regulated in all the four tissues examined at both time points, with an exceptionally high induction of 1,776-fold at 6 hpi in the liver (Figure 2G). The expression of M17 (56) was also induced but with lower fold-induction. In contrast, CNTF (56) expression was refractory after YRF injection in all tissues examined (Table 1).

IL-34 is a ligand for macrophage colony-stimulating factor-1 receptor, promotes the differentiation, proliferation, and survival of monocytes, macrophages, and osteoclasts (57). Its expression was also rapidly activated in spleen, liver and skin but only at 6 hpi (Table 1).

#### The Expression of CXC Chemokines

**Figure 3:**
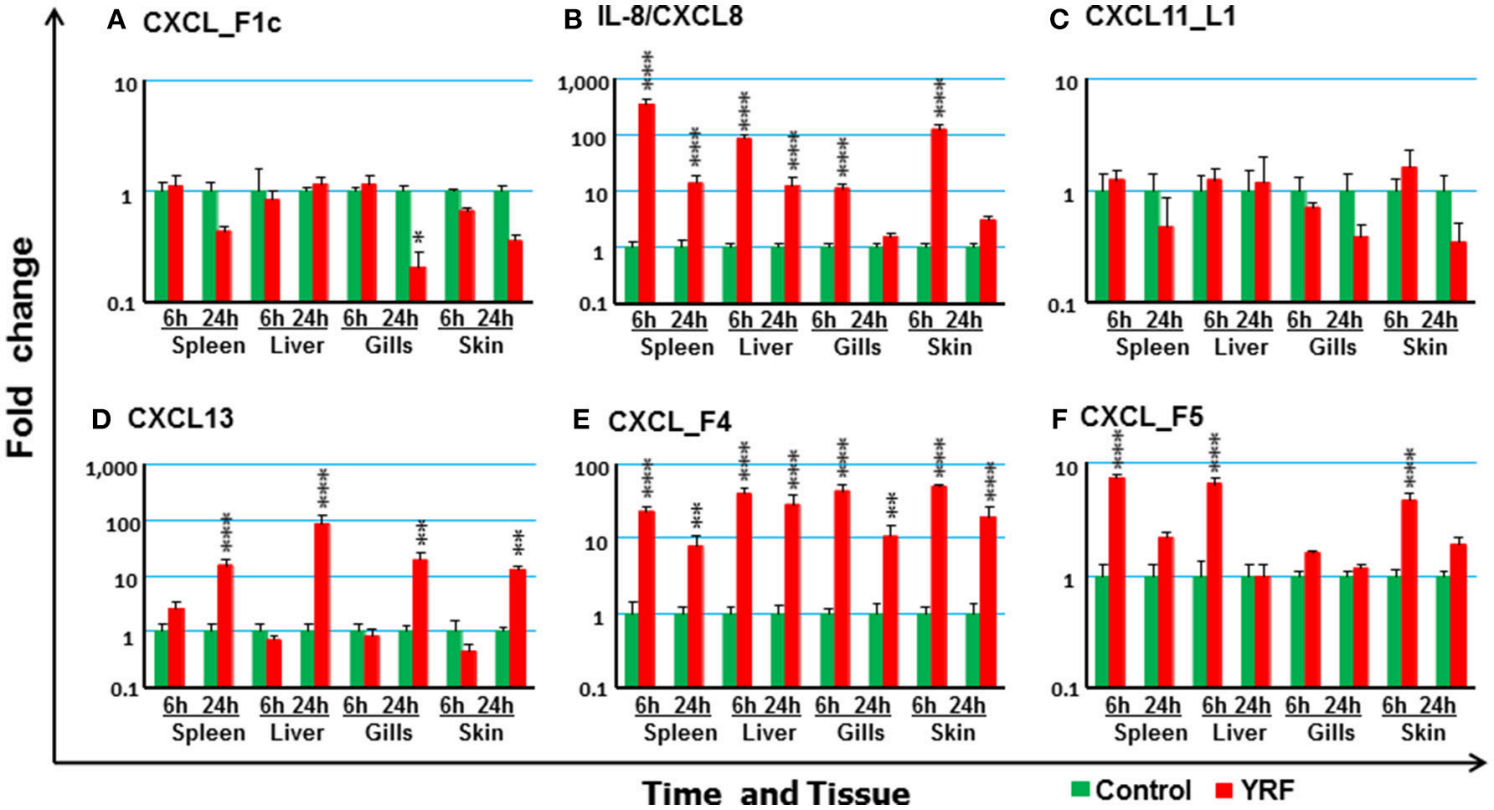
The modulation of the expression of CXC chemokines *in vivo* by YRF. Rainbow trout were ip injected with YRF or PBS, sampled and analyzed as described in Figure 2. The expression of CXCL_F1c **(A)**, IL-8/CXCL8 **(B)**, CXCL11_L1/γIP **(C)**, CXCL13 **(D)**, CXCL_F4 **(E)**, and CXCL_F5 **(F)** was examined. A fold change was calculated as the average expression level of YRF injected fish divided by that of time matched controls. The means + SEM of six fish are shown. Significant differences between injected fish and controls were tested by one way-ANOVA followed by the Bonferroni *post hoc* test and are shown over the bars as; ^*^p < 0.05, ^**^p < 0.01, and ^***^p < 0.001.

Chemokines are chemotactic cytokines that regulate cell trafficking during an immune response to recruit cells of the immune system to a site of infection. The expression of 12 known trout CXC chemokines (58) was examined. The proinflammatory CXCL8/IL-8 expression was rapidly up-regulated in the spleen, liver, gills and skin at 6 hpi, with up-regulation sustained in the spleen and liver to 24 h (Figure 3B). CXCL_F4 expression was also rapidly induced at 6 hpi and sustained to 24 hpi in all the four tissues examined (Figure 3E). A transient up-regulation of CXCL_F5 was seen in the spleen, liver and skin at the early time point (Figure 3F). However, CXCL13 expression was up-regulated at only the late time point in all tissues (Figure 3D). In contrast, CXCL_F1c expression was downregulated in gills at 24 hpi (Figure 3A). The expression of CXCL_F1a in the spleen was irregular and no significant difference was observed post YRF injection. The expression of other CXC chemokines, CXCL_F1b, CXCL_F2, CXCL11_L1/γIP, CXCL12, and CXCL14, was refractory (Figure 3C and Table 1).

#### The Expression of IL-12 Family

**Figure 4:**
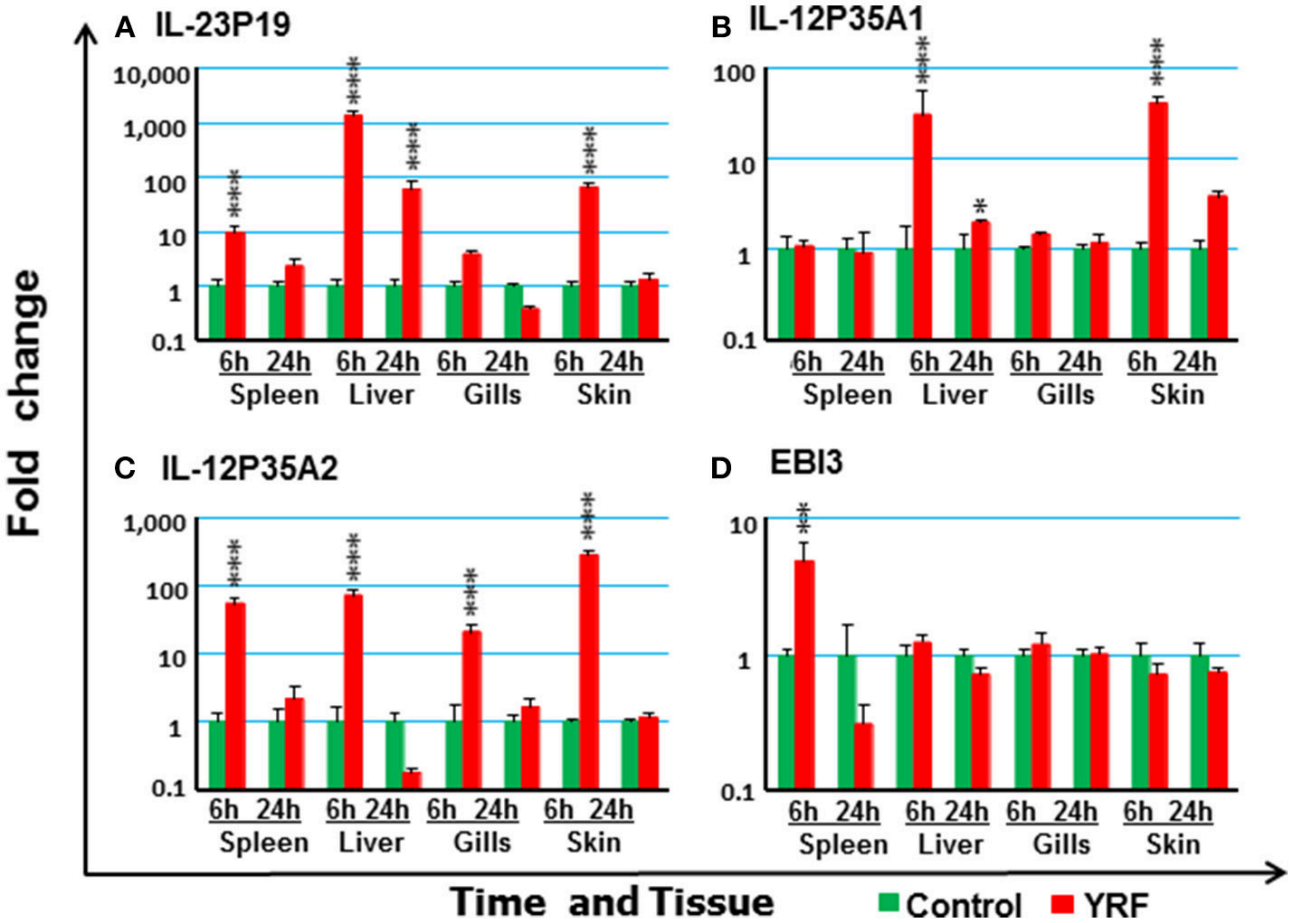
The modulation of the expression of IL-12 family cytokines *in vivo* by YRF. Rainbow trout were ip injected with YRF or PBS, sampled and analyzed as described in Figure 2. The expression of IL-23P19 **(A)**, IL-12P35A1 **(B)**, IL-12P35A2 **(C)**, and EBI3 **(D)** was examined. A fold change was calculated as the average expression level of YRF injected fish divided by that of time matched controls. The means + SEM of six fish are shown. Significant differences between injected fish and controls were tested by one way-ANOVA followed by the Bonferroni *post hoc* test and are shown over the bars as; ^*^p < 0.05, ^**^p < 0.01, and ^***^p < 0.001.

IL-12 family members are heterodimeric cytokines of α/β chains. Genes for 6 active α-chains (p19, p28A, p28B, p35A1, p35A2, and p35B) and 4 β-chains (p40B1, p40B2, p40C, and EBI3) have been cloned in rainbow trout recently (44, 46, 59, 60). P19 (59) expression was rapidly up-regulated at 6 hpi in the spleen, liver and skin, and this was sustained in liver to 24 hpi (Figure 4A). P35A1 (46) expression was only up-regulated in the liver at 6 h and 24 h and in the skin at 6 hpi (Figure 4B). A transient up-regulation was also seen with p35A2 in the spleen, liver, gills and skin, and with EBI3 (61) in spleen (Figures 4C,D). The expression of P28A (62), P28B and P40B1 was undetectable in control samples of some tissues thus a fold change could not be calculated. However, P40B1 expression was detected in the spleen and liver samples at 6 hpi. No significant change was observed in the expression of p28A, p28B, P40B2, and P40C (Table 1). These expression profiles suggest specific IL-12, IL-23 and IL-35 isoforms may be induced by *in vivo* YRF administration.

#### The Expression of Adaptive Cytokines

**Figure 5:**
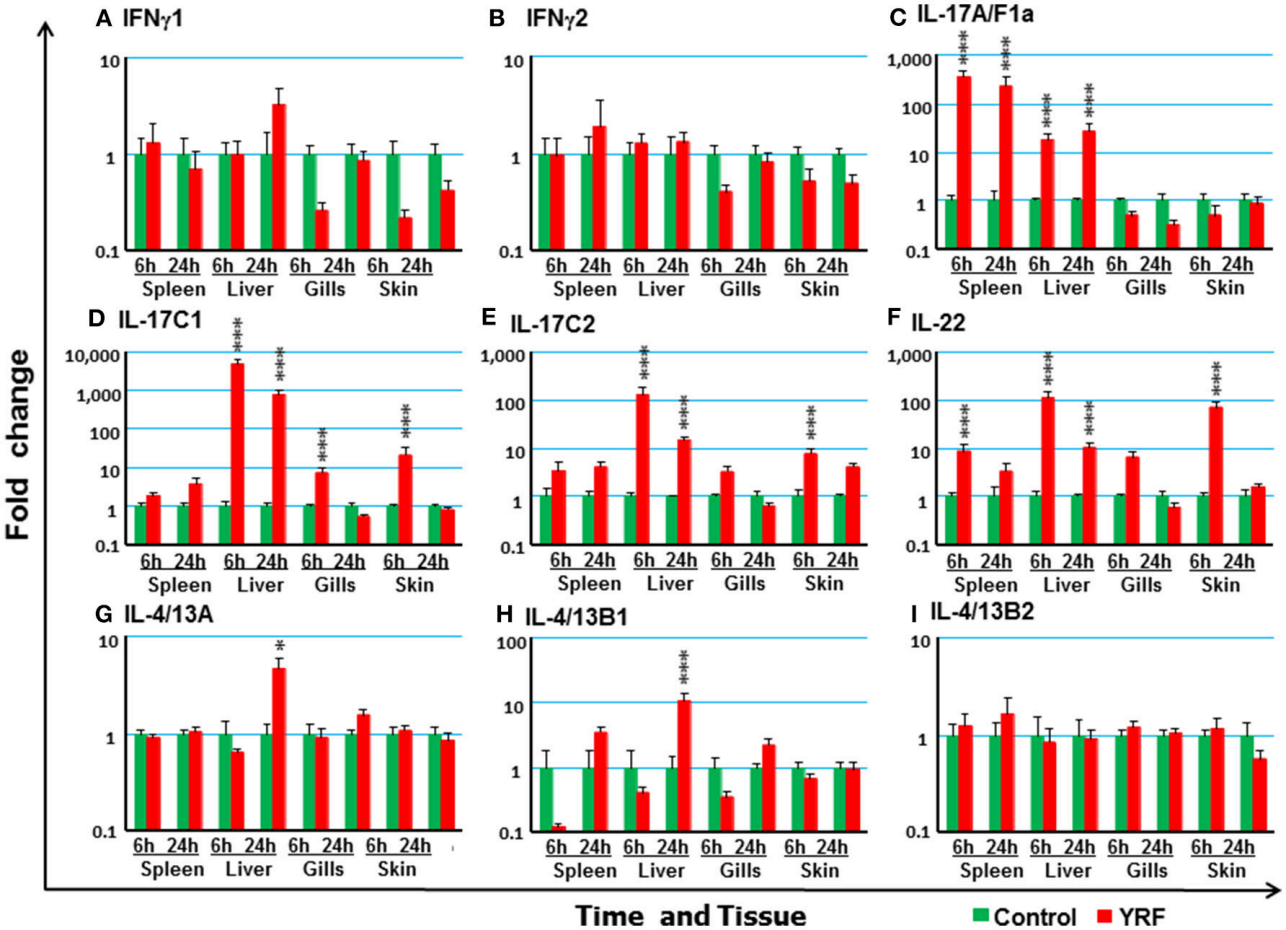
The modulation of the expression of adaptive cytokines *in vivo* by YRF. Rainbow trout were ip injected with YRF or PBS, sampled and analyzed as described in Figure 2. The expression of IFNγ1 **(A)**, IFNγ2 **(B)**, IL-17A/F1a **(C)**, IL-17C1 **(D)**, IL-17C2 **(E)**, IL-22 **(F)**, IL-4/13A **(G)**, IL-4/13B1 **(H)**, and IL-4/13B2 (**I**), was examined. A fold change was calculated as the average expression level of YRF injected fish divided by that of time matched controls. The means + SEM of six fish are shown. Significant differences between injected fish and controls were tested by one way-ANOVA followed by the Bonferroni *post hoc* test and are shown over the bars as; ^*^p < 0.05 and ^***^p < 0.001.

Next the expression of cytokines involved in adaptive immunity was examined, including IFNγ, IL-17 family, γ-chain cytokine family and IL-20/22 to investigate if they were modulated by YRF. Both IFNγ1 and IFNγ2 were refractory to YRF stimulation in all tissues (Figures 5A,B). IL-17A/F1a (63) expression was highly up-regulated in the spleen and liver at 6 hpi and 24 hpi, but not in the gills and skin at both time points post YRF injection (Figure 5C). IL-17A/F2a (64) expression was only upregulated in the liver at 24 hpi (Table 1). Interesting, IL-17C1 (65) was highly induced in the liver at 6 hpi and 24 hpi, and to a lesser extent in the gills and skin at 6 hpi (Figure 5D). Similarly, IL-17C2 expression was also induced in the liver at 6 hpi and 24 hpi and in the skin at 6 hpi (Figure 5E). The expression of IL-1A/F1b, IL-17A/F2b, IL-17A/F3, IL-17N, and IL-17D was refractory (Table 1). IL-22 (66) expression was rapidly induced in the spleen, liver, gills and skin at 6 hpi, and this was sustained in the liver to 24 hpi (Figure 5F). IL-20L (62) expression was refractory (Table 1).

Upregulation of Th2 cytokines (67) was seen only in the liver at 24 hpi, for IL-4/13A and IL-4/13B1 but not IL-4/13B2 (Figures 5G–I). IL-15 (68) expression was also seen up-regulated in the liver at 6 and 24 hpi, and in the gills at 6 hpi (Table 1). The expression of other γ-chain cytokines, IL-2 (69) and IL-21 (47) was refractory in all tissues (Table 1).

#### The Expression of Anti-inflammatory Cytokines

**Figure 6:**
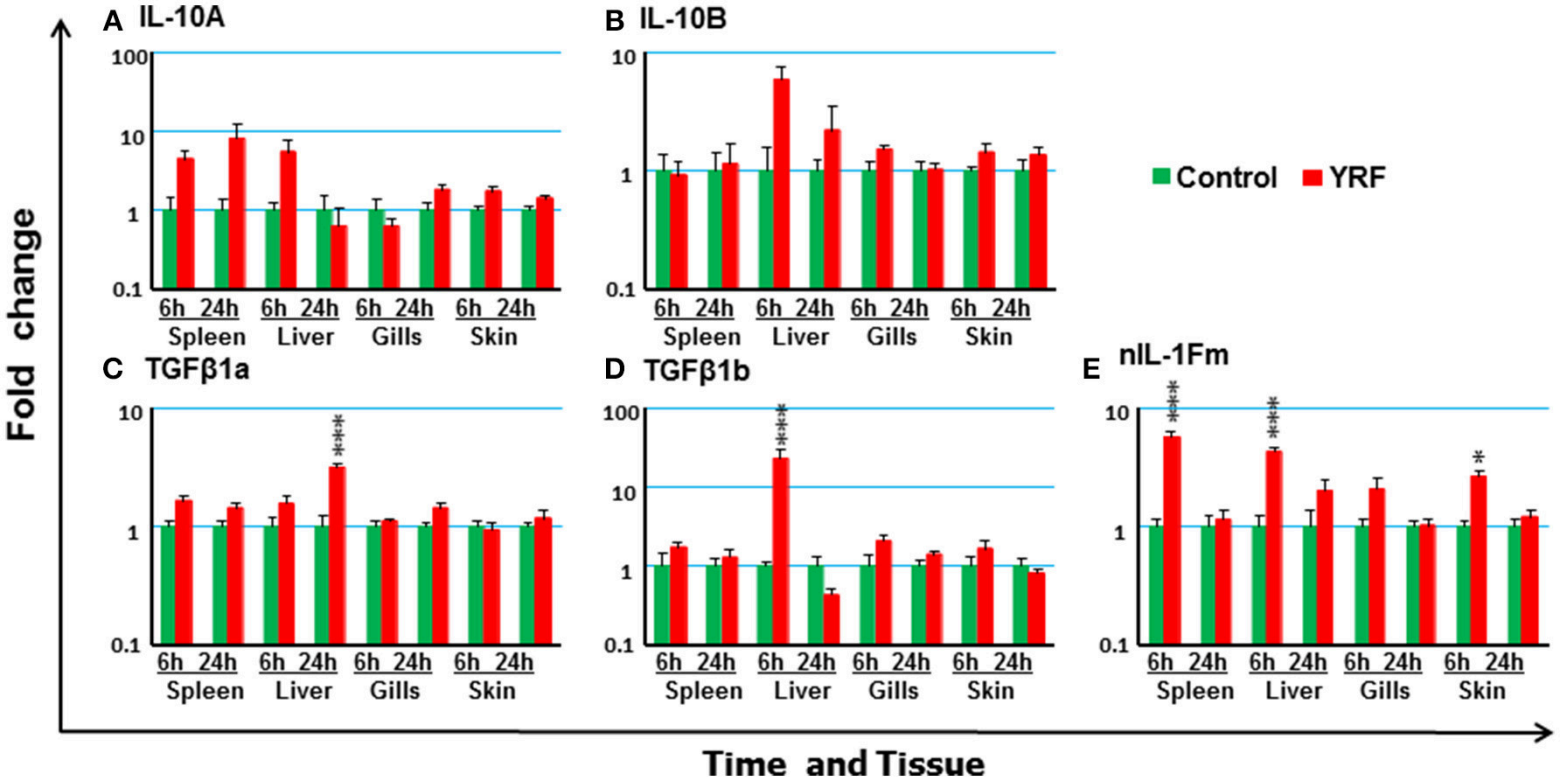
The modulation of the expression of anti-inflammatory cytokines genes *in vivo* by YRF. Rainbow trout were ip injected with YRF or PBS, sampled and analyzed as described in Figure 2. The expression of IL-10A **(A)**, IL-10B **(B)**, TGFβ1a **(C)**, TGFβ1b **(D)**, and nIL-1Fm **(E)** was examined. A fold change was calculated as the average expression level of YRF injected fish divided by that of time matched controls. The means + SEM of six fish are shown. Significant differences between injected fish and controls were tested by one way-ANOVA followed by the Bonferroni *post hoc* test, and are shown over the bars as; ^*^p < 0.05 and ^***^p < 0.001.

The rapid but transient activation of major proinflammatory cytokine gene expression suggests some negative regulatory mechanisms must be activated by YRF. Thus, the expression of several anti-inflammatory cytokines known in rainbow trout was examined post YRF injection. The expression of both IL-10A and IL-10B (70) was refractory (Figures 6A,B). A small induction was seen in the liver at 24 hpi for TGFβ1A and at 6 hpi for TGFβ1B (71) (Figures 6C,D). In addition, the expression of nIL-1Fm was induced in the spleen, liver and skin at 6 hpi (Figure 6E).

#### The Expression of SOCS Genes

**Figure 7:**
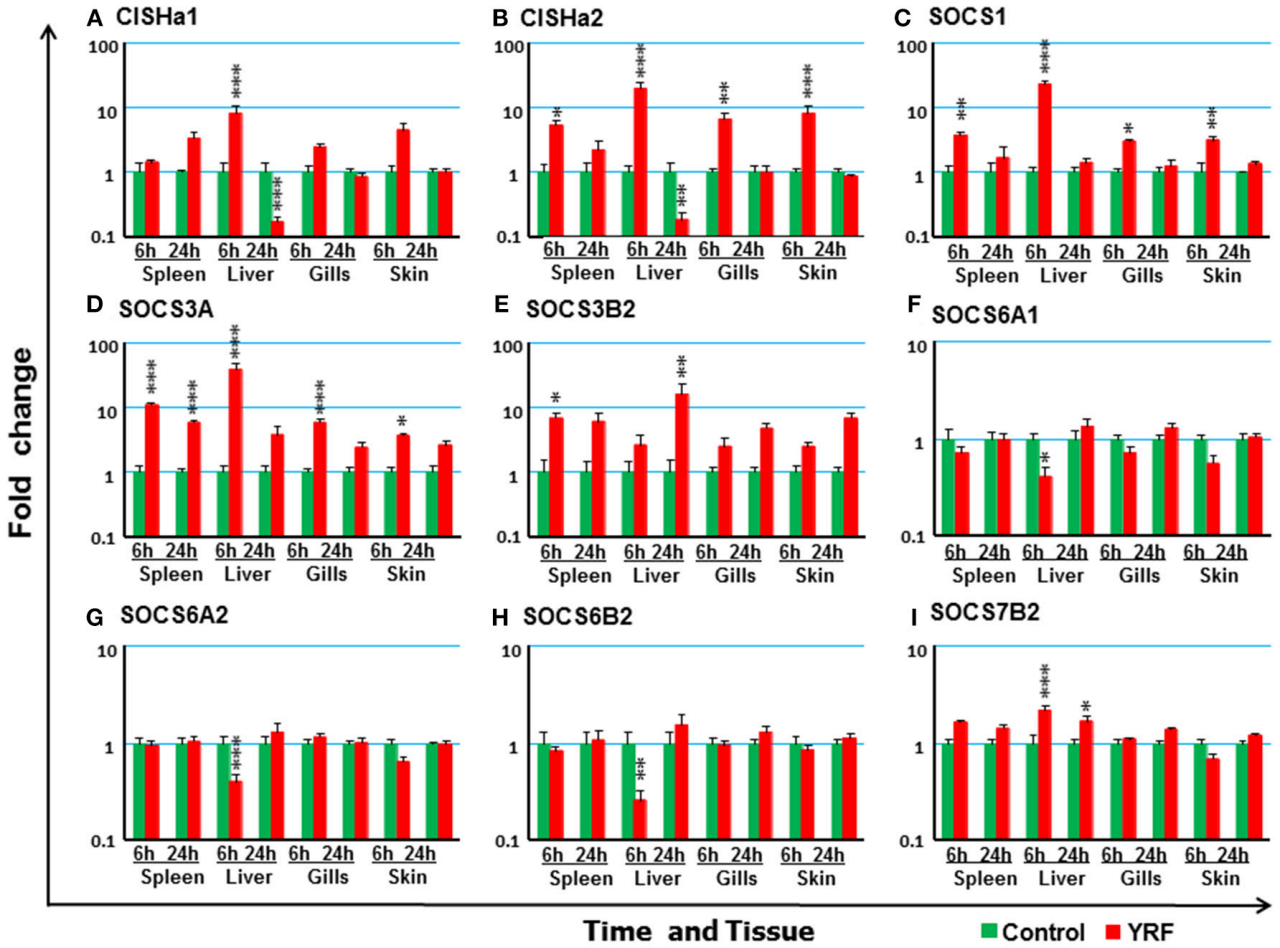
The modulation of the expression of SOCS genes *in vivo* by YRF. Rainbow trout were ip injected with YRF or PBS, sampled and analyzed as described in Figure 2. The expression of CISHa1 **(A)**, CISHa2 **(B)**, SOCS1 **(C)**, SOCS3A **(D)**, SOCS3B2 **(E)**, SOCS6A1 **(F)** SOCS6A2 **(G)** SOCS6B2 **(H)** SOCS7B2 **(I)** was examined. A fold change was calculated as the average expression level of YRF injected fish divided by that of time matched controls. The means + SEM of six fish are shown. Significant differences between injected fish and controls were tested by one way-ANOVA followed by the Bonferroni *post hoc* test, and are shown over the bars as; ^*^p < 0.05, ^**^p < 0.01, and ^***^p < 0.001.

The SOCS family are key negative regulators of cytokine and growth factor signaling, with 26 SOCS genes expressed in rainbow trout [including an expressed pseudogene, (72)]. CISHa1 (73) was upregulated at 6 hpi but downregulated at 24 hpi in the liver (Figure 7A). CISHa2 was also up-regulated at 6 hpi and downregulated at 24 hpi in the liver, but in addition it was upregulated at 6 hpi in the spleen, gills and skin (Figure 7B). Both SOCS1 and SOCS3A were rapidly upregulated at 6 hpi, but this had subsided at 24 hpi in all four tissues examined with the exception of SOCS3A in the spleen (Figures 7C,D). SOCS3B2 expression was only increased in spleen at 6 hpi and in liver at 24 hpi (Figure 7E). The expression of both SOCS6A1, SOCS6A2, and SOCS6B2 decreased at 6 hpi in the liver (Figures 7F–H and Table 1). SOCS7B2 was increased in the liver at 6 and 24 hpi (Figure 7I). The expression of other SOCS genes, CISHb1-2, SOCS1B, SOCS2A1-2, SOCS2B, SOCS3B1, SOCS4, SOCS5A1-2/B1-2, SOCS6B1, and SOCS7A1-2, was refractory in all tissues (Table 1).

### Systematic Activation of Anti-microbial Defense Pathways *in vivo* by YRF

**Figure 8:**
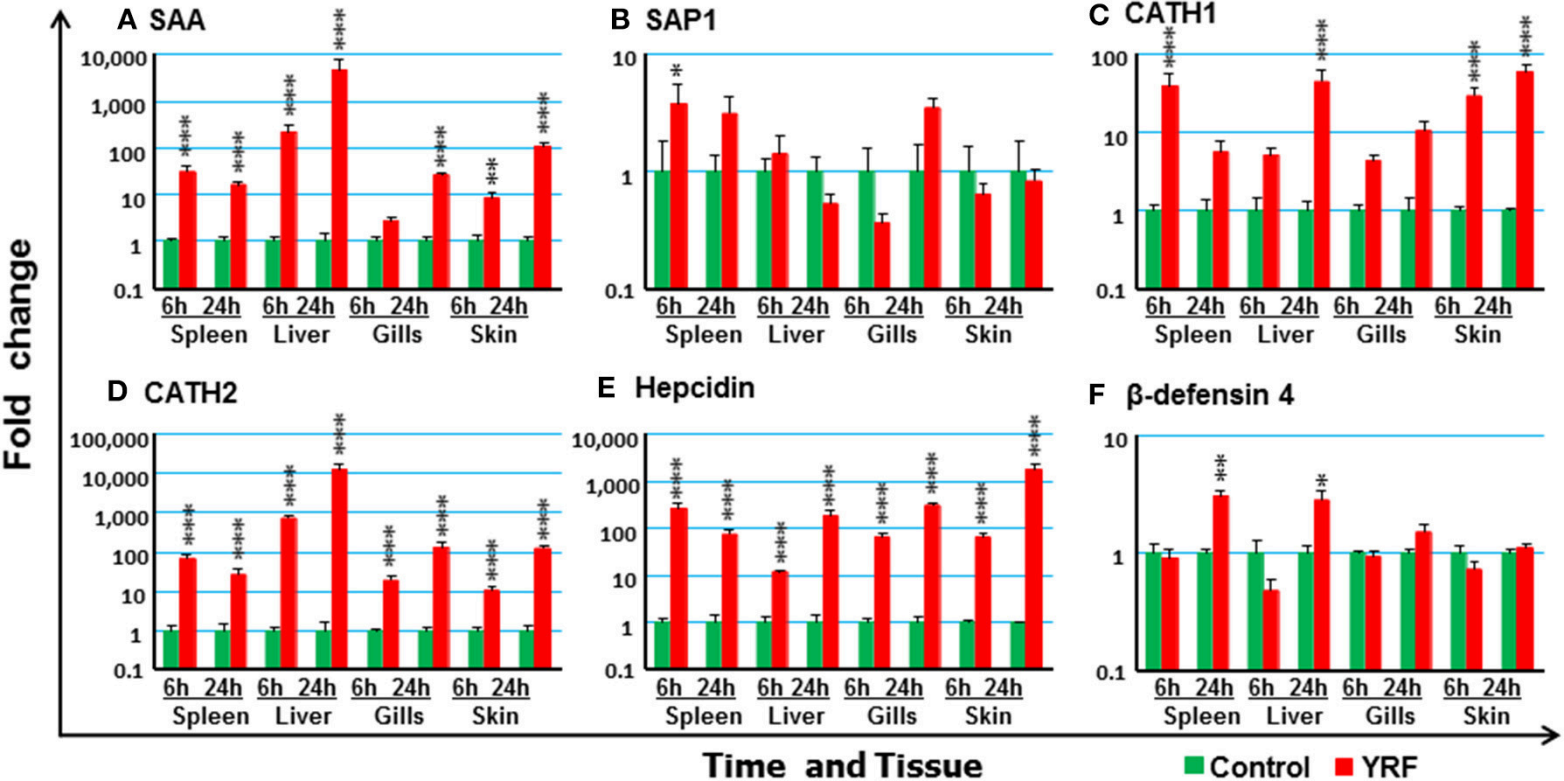
The activation of APPs and AMPs *in vivo* by YRF. Rainbow trout were ip injected with YRF or PBS, sampled and analyzed as described in Figure 2. The expression of SAA **(A)**, SAP1 **(B)**, CATH1 **(C)**, CATH2 **(D)**, hepcidin **(E)**, and β-defensin-4 **(F)**, was examined. A fold change was calculated as the average expression level of YRF injected fish divided by that of time matched controls. The means + SEM of six fish are shown. Significant differences between injected fish and controls were tested by one way-ANOVA followed by the Bonferroni *post hoc* test, and are shown over the bars as; ^*^p < 0.05, ^**^p < 0.01, and ^***^p < 0.001.

To investigate potential antimicrobial pathways activated by YRF injection, the expression of several known APPs, AMPs and complement components was examined. SAA (74) expression was upregulated in the spleen, liver and skin at 6 hpi, and in the spleen, liver, gills and skin at 24 hpi (Figure 8A). SAP1 expression was upregulated only in the spleen at 6 hpi (Figure 8B), and SAP2 was refractory (Table 1).

A systemic activation of cathelicidins, hepcidin and β-defensin 4 was observed in multiple tissues after YRF administration (Figures 8C–F). CATH1 (75) expression was up-regulated in the spleen, liver and skin at 6 hpi, and this was sustained in the skin to 24 hpi (Figure 8C). The expression of CATH2 and hepcidin (76) was induced and sustained from 6 hpi to 24 hpi in all the four tissues examined (Figures 8D–E). It is noteworthy that the induction of CATH2 and hepcidin in the liver, gills and skin was higher at 24 hpi than that at 6 hpi. A small induction of β-defensin 4 (77) expression was observed in the spleen and liver but only at 24 hpi (Figure 8F), whilst the expression of β-defensin 1–3 was refractory (Table 1). The expression of both LEAP2A and LEAP2B was down-regulated in the liver at 24 hpi (Table 1).

### The Expression of Complement Genes *in vivo* After YRF Injection

**Figure 9:**
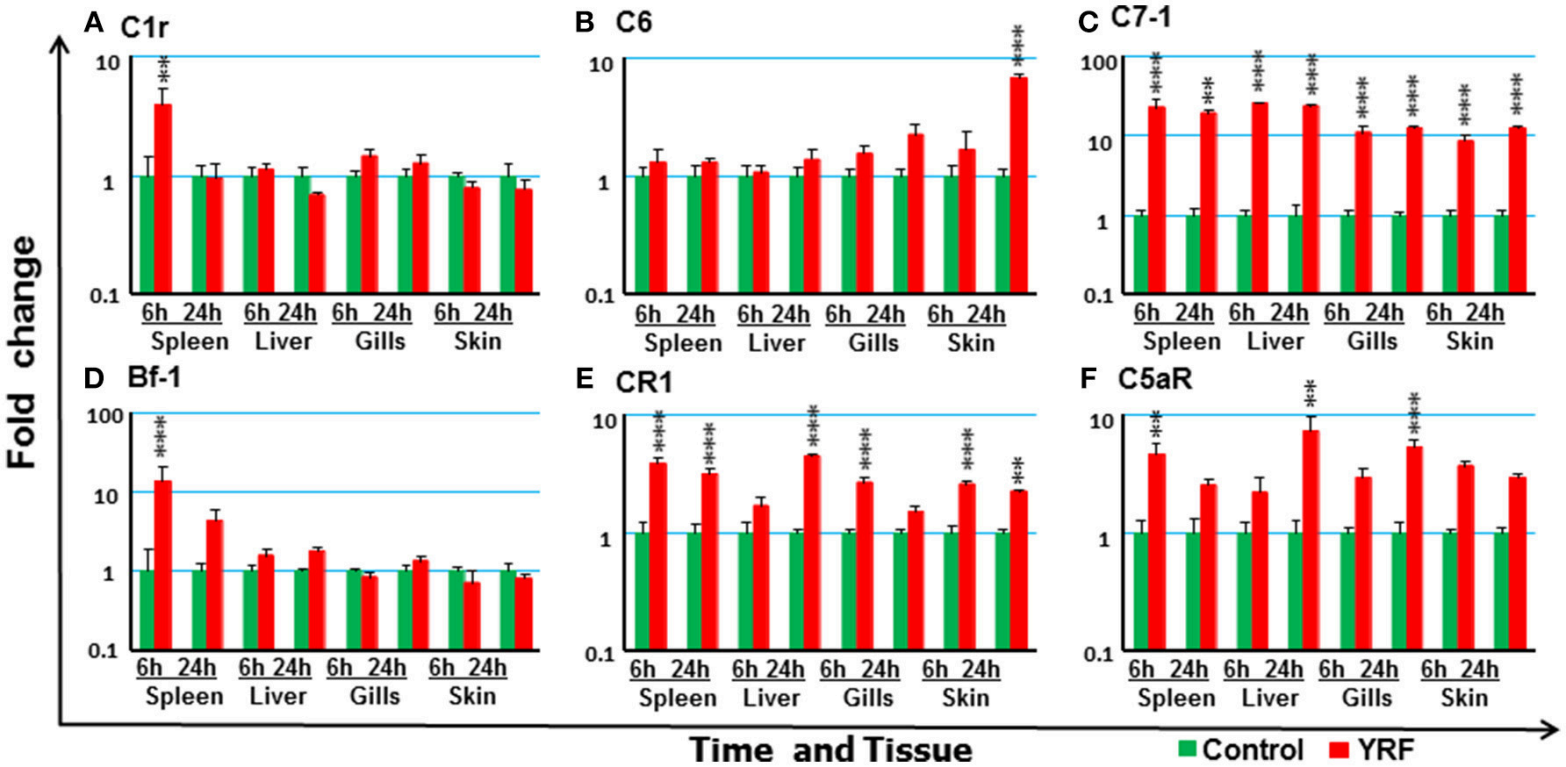
The activation of complements *in vivo* by YRF. Rainbow trout were ip injected with YRF or PBS, sampled and analyzed as described in Figure 2. The expression of complement genes C1r **(A)**, C6 **(B)**, C7-1 **(C)**, Bf-1 **(D)**, CR1 like **(E)**, and C5aR **(F)** was examined. A fold change was calculated as the average expression level of YRF injected fish divided by that of time matched controls. The means + SEM of six fish are shown. Significant differences between injected fish and controls were tested by one way-ANOVA followed by the Bonferroni *post hoc* test, and are shown over the bars as; ^**^p < 0.01 and ^***^p < 0.001.

The expression of 18 trout genes in the complement system (78) was examined after YRF injection (Figure 9). C7-1 expression was activated and sustained from 6 to 24 hpi in all four tissues examined (Figure 9C). The expression of C1r and Bf-2 was increased in spleen at 6 hpi (Figures 9A,D). C6 expression was increased in the skin but only at 24 hpi (Figure 9B). Whilst the expression of complement receptor CR1-like gene was increased at 6 and 24 h hpi in both spleen and skin, it was only increased at 6 hpi in the gills and at 24 hpi in the liver (Figure 9E), C5aR expression was increased at 6 hpi in the spleen, and at 24 hpi in the liver and gills (Figure 9F). The expression of other complement genes tested was refractory (Table 1)

### Modulation of the Expression of Arginase Genes and TLR5 *in vivo* by YRF

**Figure 10:**
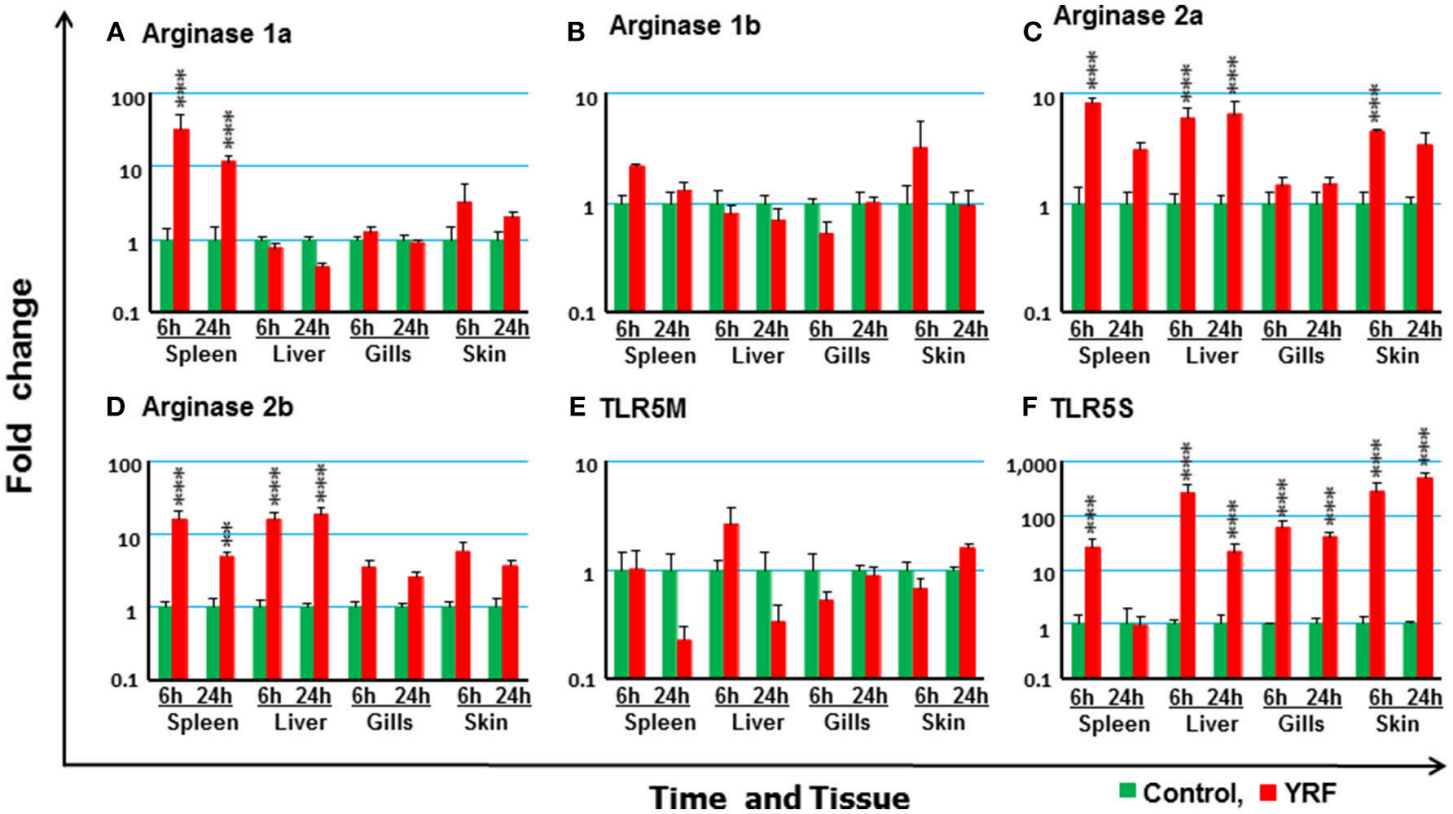
The modulation of the expression of arginase genes and TLR5 *in vivo* by YRF. Rainbow trout were ip injected with YRF or PBS, sampled and analyzed as described in Figure 2. The expression of arginase 1a **(A)**, arginase 1b **(B)**, arginase 2a **(C)**, arginase 2b **(D)**, TLR5M **(E)**, and TLR5S **(F)** in each tissue was examined by RT-qPCR. A fold change was calculated as the average expression level of YRF injected fish divided by that of time matched controls. The means + SEM of six fish are shown. Significant differences between injected fish and controls were tested by one way-ANOVA followed by the Bonferroni *post hoc* test, and are shown over the bars as; ^**^p < 0.01 and ^***^p < 0.001.

Macrophages are a first line of innate responders controlling and organizing host defenses against pathogens and are essential for maintaining homeostasis (79). They undergo specific activation into functionally distinct phenotypes, such as M1 and M2 (80). Arginase expression is an important marker of macrophage activation, with four genes present in rainbow trout (81). Whilst the expression of arginase 1a was upregulated at 6 and 24 hpi in the spleen, arginase 1b expression was refractory (Figures 10A,B). Arginase 2a expression was rapidly activated in the spleen, liver and skin at 6 hpi, and this was sustained in the liver to 24 hpi (Figure 10C). Arginase 2b expression was upregulated in both spleen and liver at 6 and 24 hpi (Figure 10D).

To further study the molecular mechanisms involved in the above induction of immune genes by flagellin, the two types of TLR5 genes (TLR5M and TLR5S, 24) present in trout were examined. The expression of TLR5M and TLR5S was detectable in all the tissues. However, there was no significant impact of YRF on the expression of TLR5M in any tissue (Figure 10E). Interestingly, TLR5S expression was significantly up-regulated in all tissues at 6 hpi and this was maintained to 24 hpi in liver, gills and skin (Figure 10F).

### Clustering Analysis of Genes Modulated by YRF *in vivo*

**Figure 11:**
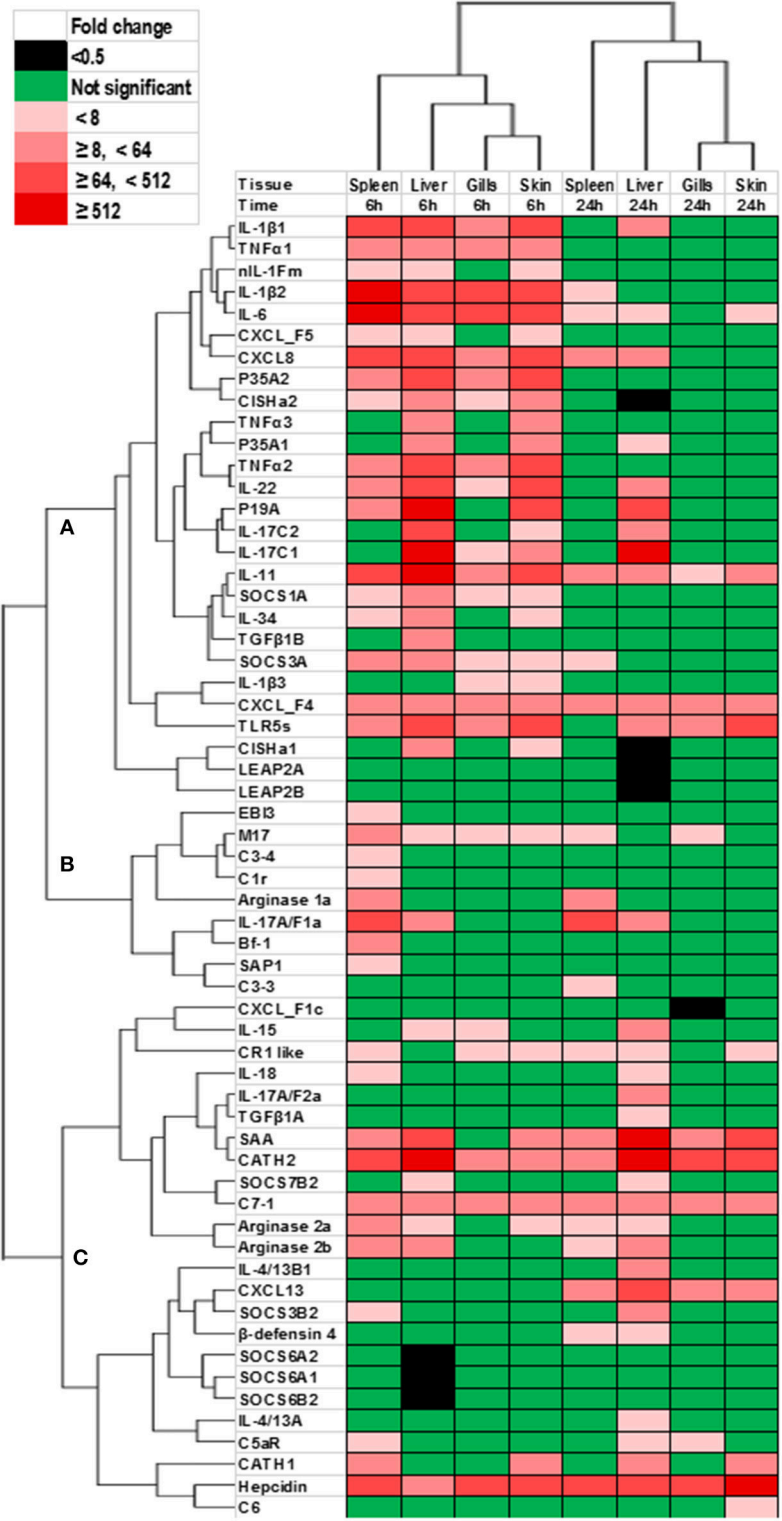
Clustering analysis of genes modulated by YRF *in vivo*. Rainbow trout were ip injected with YRF or PBS, sampled and gene expression analyzed. Genes significantly modulated (at least onetime point/tissue) were selected for analysis. The fold changes were log2 transformed and subjected to hierarchical clustering using the Morpheus program. Three major expression profiles (clusters **A–C**) of genes are apparent.

The genes that showed significant changes after YRF injection were subjected to hierarchical clustering analysis that revealed a time effect upon gene expression, with the gills and skin showing a similar response (Figure 11). Three major expression profiles (clusters A-C) of genes were observed after YRF injection. Cluster A included the major proinflammatory cytokines (IL-1β, TNFα, IL-6, IL-11, IL-17C, IL-12P35A, and IL-23P19), chemokines (CXCL8 and CXCL_F4-5), anti-inflammatory genes (TGFβ1B, CISHa, SOCS-1A, and SOCS3A) and TLR5S. These genes were rapidly induced at 6 hpi by YRF in all or most of the tissues but had decreased at 24 hpi (Figures 2–7, 10 for detail), suggesting rapid albeit transient activation. Cluster B, including IL-17A/F1a, EBI3, arginase-1 and several complement genes, was also rapidly induced but mainly in the spleen. Cluster C included the cytokines IL-4/13, IL-15, IL-17A/F2a, IL-18, and TGFβ1a, arginase-2, several complement genes and SOCS genes, APP and AMPs (Figure 11). This cluster of genes showed higher expression at 24 hpi, especially in the liver.

### Antibody Response to YRF Vaccination in Rainbow Trout

**Figure 12:**
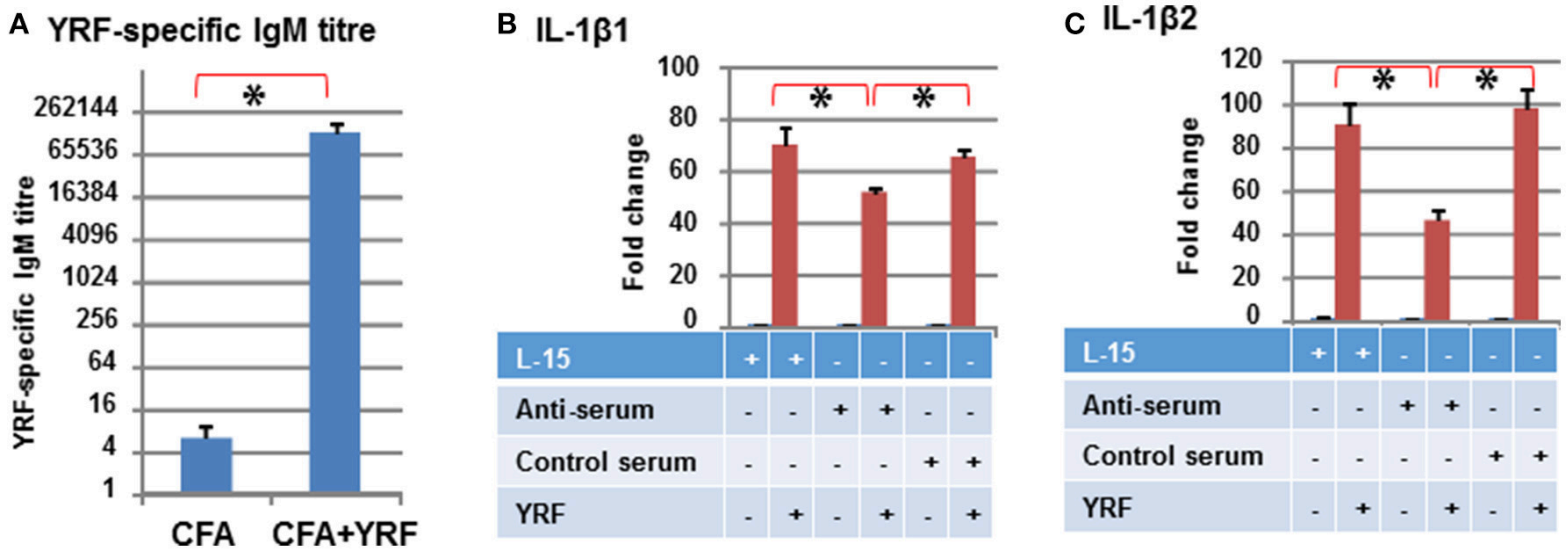
Antibody response to YRF vaccination. **(A)** YRF-specific IgM antibody titer. Rainbow trout were immunized with CFA+YRF or CFA alone as control. YRF specific IgM antibody was detected by ELISA in sera collected 3 months post immunization. The mean+SEM of 10 fish from each group are shown. Neutralization of YRF activated IL-1β1 **(B)** and IL-1β2 **(C)** expression. YRF was incubated with sera from CFA+YRF immunized trout (Anti-serum), or sera from CFA immunized fish (Control serum), or cell culture medium (L-15) for 1 h before addition to RTS-11 cells. Matched serum or L-15 samples without YRF were also added to RTS-11 cells as controls. After 4 h the incubation was terminated and the expression of IL-1β1 and IL-1β2 determined by RT-qPCR as described above. The bridge and asterisk connected bars indicate a statistical difference (*P* < 0.05).

Low titres (average of 6) of anti-YRF IgM antibodies were detected by ELISA in the sera 3 months-post injection with CFA (Control serum). The YRF-specific IgM antibody titres were markedly increased to 130,253 (average of 10 fish, *p* < 0.001) in fish immunized with YRF/CFA (Anti-serum) (Figure 12A). When 10 ng of YRF was incubated with cell culture medium (L-15), anti-serum or control serum for 1 h, it could still significantly activate the expression of IL-1β1 and IL-1β2. However, the fold change of the anti-serum treated YRF was lower than YRF incubated with L-15 or with control serum (Figures 12B,C). These results show that YRF-specific antibodies are induced by flagellin immunization, and that the antiserum can decrease flagellin bioactivity.

### Anti YRF Antibody Reacts Against the Middle D2/D3 Domain

**Figure 13:**
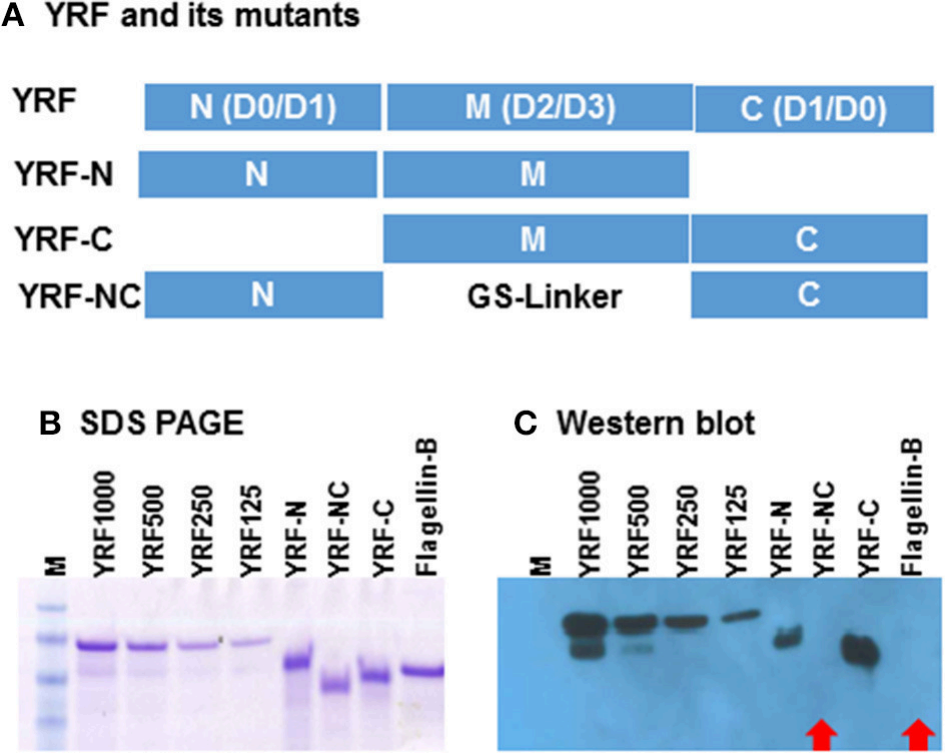
**(A)** Schematic representation of YRF and its mutants. Full-length YRF contains the N-terminal D0/D1 domains, the middle unstructured D2/D3 domain and the C terminal D1/D0 domains. Three mutants with the N-terminal deleted (YRF-C), middle domain deleted (YRF-NC) and the C-terminal deleted (YRF-N) were produced. **(B)** SDS-PAGE and **(C)** Western blot analysis of YRF and its mutants. YRF1000, 500, 250, and 125 indicate amount (ng) of protein loaded. An unrelated flagellin protein (Flagellin-B) was also included as a control. The protein gel was stained with Imperial protein stain **(B)** or detected by Western blotting **(C)** using antisera from rainbow trout immunized with YRF + CFA. M = SeeBlue plus 2 protein marker. Red arrows indicate a negative signal.

Full-length YRF contains N-terminal D0/D1 domains, middle unstructured D2/D3 domains and C terminal D0/D1 domains. To investigate the binding sites/domains of the anti-YRF antibodies produced in rainbow trout, three mutants were produced with the N-terminal deleted (YRF-C), middle domain deleted (YRF-NC) and the C-terminal deleted (YRF-N) (Figure 13A). The YRF and its mutants had expected sizes of 45.4, 33.6, 29.2 and 30.9 kDa for YRF, YRF-N, YRF-NC, and YRF-C, respectively, and were successfully produced as shown by SDS-PAGE after Imperial protein staining (Figure 13B). Western blot analysis revealed that the antibodies produced after YRF immunization reacted with full-length YRF, as well as the mutant proteins with the middle D2/D3 domain retained (ie YRF-N and YRF-C), but lost the reactivity against YRF-NC with the middle D2/D3 domains deleted. Furthermore, the antiserum did not react against an unrelated flagellin (flagellin-B) from an unrelated fish bacterial pathogen (Figure 13C). This data shows that the middle D2/D3 domain of YRF contains the major antigenic determinants recognized by the host (rainbow trout), and that the his-tag (ASSAHHHHHHHHHH) did not contribute significantly to YRF immunogenicity.

### Mutation of YRF Abrogates YRF Proinflammatory Activity

**Figure 14:**
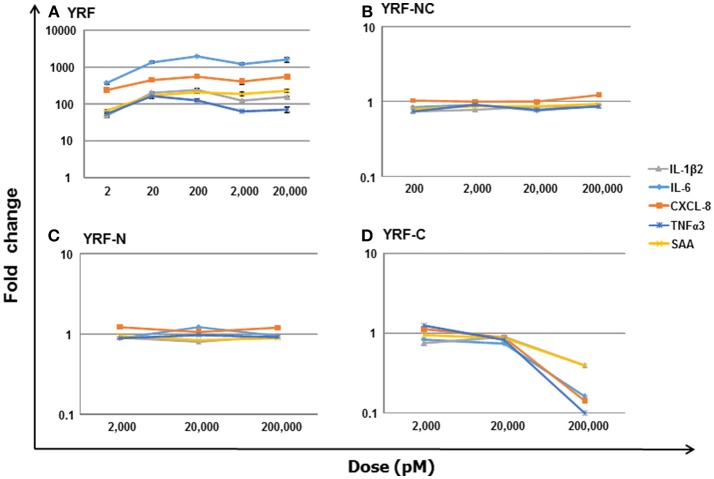
Dose-response of biomarker gene expression in response to YRF and its mutants. RTS-11 cells was stimulated with different concentrations of YRF **(A)**, and its mutants YRF-NC **(B)**, YRF-N **(C)**, and YRF-C **(D)** for 4 h. The gene expression of IL-1β2, IL-6, CXCL-8, TNFα3, and SAA was determined by qPCR, and expressed as a fold change relative to the control samples. The means + SEM of four independent samples are shown. Note: the expression of all the genes was significantly different from controls in YRF stimulated samples only.

To directly compare the proinflammatory activity of YRF and its mutants, a dose-response analysis on a molar basis was undertaken, using RTS-11 cells stimulated for 4 h. An initial analysis revealed that the mutants lost their ability to induce inflammatory gene expression. Thus, different (increased) dose ranges were chosen, e.g,. 2–20,000 pM for YRF, 2,000–200,000 pM for YRF-N and YRF-C, and 200-200,000 pM for YRF-NC. As expected, YRF was able to upregulate the expression of all genes tested, including IL-1β2, IL-6, CXCL-8, TNFα3, and SAA at all doses tested (2–20,000 pM) (Figure 14A). However, all the YRF mutants lost the ability to upregulate the biomarker gene expression at all the doses tested (Figures 14B–D).

## Discussion

Using an *in vitro* fish macrophage model, we have demonstrated that, compared to LPS and peptidoglycan, flagellin is a particularly potent inflammatory trigger, in terms of activation of proinflammatory cytokine gene expression. Therefore, the immune stimulatory effects of flagellin were studied *in vivo* in an important model fish species, the rainbow trout. After ip injection with the recombinant flagellin YRF, from the fish pathogen *Yersinia ruckeri*, a transient systemic inflammation was activated, along with multiple antimicrobial pathways. Tissue-specificity of the response was also observed. High antibody titres could be induced by flagellin, and these antibodies were shown to react against the middle D2/D3 domains (demonstrated using a variety of muteins), and to neutralize the bioactivity of flagellin. Interestingly, the bioactivity was retained only with full-length YRF. These results are discussed in the context of innate host responses to flagellin, host/pathogen interactions, and the use of flagellin as an immune stimulant or vaccine adjuvant in fish aquaculture.

### A Transient Systemic Activation of Inflammation

To use a PAMP, such as a flagellin, as an immunostimulant or a vaccine adjuvant in a fish species, it is necessary to first understand the early events of the host response, the activation of inflammatory responses and anti-microbial defense mechanisms. In this study we used a targeted approach to determine the impact of YRF on selected immune relevant genes as a means to gain in-depth information on these events in our model species, the rainbow trout.

Following YRF administration multiple proinflammatory cytokines, including IL-1β, TNFα, IL-6, IL-11, M17, IL-17C, IL-12, IL-22, IL-23, and chemokines (CXCL8, and CXCL_F4-5) were induced by 6 hpi in all or most of the tissues examined but had decreased by 24 hpi. A similar response was also observed for several anti-inflammatory genes, including nIL-1Fm, IL-35 and the type II SOCS family genes (CISHa, SOCS1, and SOCS3A) that function intracellularly. However, the anti-inflammatory cytokines IL-10A and IL-10B were refractory, and TGFβ1 was only induced to a small extent in the liver although several proinflammatory cytokine genes were highly induced. These expression profiles suggest a rapid but transient systemic inflammatory host response is elicited after YRF injection.

The huge early induction (three orders of magnitude) of IL-1β2 and IL-6 in the spleen, and IL-11, IL-17C1, and IL-23P19 in liver is noteworthy. Trout IL-1β is known to induce the expression of itself and other inflammatory cytokines, eg TNF-α (51), IL-6 (52), IL-11 (55), IL-17C2 (65), IL-23P19 (61), IL-34 (57), and chemokines, eg CXCL8_L1 (IL-8), CXCL11_L1, CXCL_F4, and CXCL_F5 (58). Trout IL-6 has been shown to have effects on macrophages and B cells and promote the expression of AMP genes, eg, hepcidin, CATH2 and LEAP2, and complement genes (52), but has little effects on the expression of proinflammatory cytokines. Although there are no bioactivity studies reported for trout IL-11, IL-17C, and IL-23, it is possible to assume their function from other fish species (eg IL-17C) and mammals, although it should be noted that fish IL-17C genes may have arisen from an ancestral gene that gave rise to mammalian IL-17C and IL-17E/IL-25 (65). Hence, the expression of many of the proinflammatory cytokines and other immune genes may be attributed to the early induction of IL-1β or IL-6. However, the high level induction of IL-11, IL-17C1 and IL-23P19 expression is in the liver where IL-1β expression is moderate. Furthermore, IL-1β preferentially induces the expression of IL-17C2 but not IL-17C1 that is highly induced by YRF. These expression profiles may suggest multiple cells or signaling pathways are activated by YRF injection in different tissues.

The lack of response of Th1 pathway genes (82) in all tissues was surprising. This included IFNγ1, IFNγ2, and IFNγ inducible chemokine CXCL11_L (58), and the cytokines IL-2 and IL-21 that induce IFNγ expression (47, 69). However, the Th17 marker genes IL-17A/F1 and IL-17A/F2 (63) were induced in spleen and/or liver, whilst the Th2 cytokines IL-4/13A and IL-4/13B (67) and Treg pathway cytokine TGFβ1 were induced in liver only. The implications of these responses will be discussed later in terms of the potential adjuvant effects of flagellin.

### Systemic Activation of Multiple Anti-microbial Defense Pathways by IP Injection of YRF

In response to flagellin injection, fish rapidly increase the expression of multiple APPs, AMPs and complement genes in addition to proinflammatory cytokines and chemokines. These molecules may play an important role in mediating protection against intruding pathogens. Cytokines such as IL-1β and IL-6 induce the expression of APPs, AMPs and complement genes, and may indirectly induce their upregulation following YRF injection.

APPs are involved in diverse defensive functions, such as induction of cytokine synthesis, leukocyte recruitment, activation of epithelial immunity, opsonisation and antiviral activity. Trout SAA is strongly induced in a wide variety of immune-relevant tissues by infection or challenge with PAMPs and can be detected in all primary defense barriers and in mononuclear cells of head kidney, spleen and liver, suggesting a role in local defense (74). AMPs, such as hepcidins, cathelicidins and β-defensins play an important role in defense against microbial invasion (75–77). Although the primary mode of action of antimicrobial peptides has been described as lysis of pathogens, they have also been reported to exert a number of other effects such as immune modulation. For example, mammalian hepcidin regulates iron homeostasis, and cathelicidins have been implicated in wound healing, angiogenesis, and other innate immune mechanisms (52). The complement system plays a central role in early pathogen defense by opsonization of pathogens, release of anaphylatoxins, and formation of the membrane attack complex (78). Interestingly, the peak induction of APPs, AMPs and complement genes was delayed, with highest expression observed at 24 hpi in the liver, gills and skin. The expression modulation patterns suggest that YRF injection activates an early inflammation leading to activation of systemic antimicrobial defenses that may account for the non-specific protection seen in rainbow trout against different bacteria after YRF injection (36). Thus flagellin may have potential to be used as an immunostimulant in fish aquaculture.

Interestingly, arginase 1 was up-regulated in only spleen whilst arginase 2 was induced in the spleen, liver and skin. In mammals, macrophages have functionally distinct phenotypes defined by two activation states, M1 and M2, which represent two polar ends of a continuum exhibiting pro-inflammatory and tissue repair activities, respectively (80). Arginase is one of the key components driving the molecular mechanisms involved in macrophage polarization. Arginase 1 is induced by the Th2 cytokines IL-4 and IL-13, and is considered a M2 marker in mammals (80). However, arginase 2 may be a more relevant marker of M2 cells in teleost fish (81). In rainbow trout arginase 1 expression is highly modulated by PAMPs and pro-inflammatory cytokines whilst arginase 2 is induced by the fish specific Th2 cytokine IL-4/13 (81). Whether the increased expression of arginases is due to increased trafficking of arginase expressing macrophages, or is induced *in situ* directly by flagellin or by upregulated cytokines, or both, remains to be determined. The co-expression of arginase 2 and IL-4/13 in the liver may suggest the activation of a homeostatic tissue repair mechanism after an acute inflammatory event.

### Tissue Specific Responses to IP Injection of YRF

The clustering analysis revealed a tissue specific gene expression profile, with the liver showing modulation of the largest number of immune genes, especially at 24 hpi. The liver is the central metabolic organ that must actively prevent the induction of immune responses to gut-derived antigens, and neo-antigens that are generated by the liver's metabolic and detoxification activities. In addition, gut-derived microbes and microbial products must be removed from the circulation to prevent systemic immune activation. Thus, the liver serves as an important barrier between the gut and the circulation. Both the maintenance of hepatic tolerance and the initiation of inflammation are mainly mediated by the liver-resident antigen-presenting cells (APCs) that are constantly exposed to gut-derived dietary and microbial antigens (83). These APCs include liver-resident dendritic cells, liver-resident macrophages, Kupffer cells and the liver sinusoidal endothelial cells, and semi-professional APCs, such as hepatocytes and hepatic stellate cells. These cells are instrumental for the downregulation of immune responses in the liver and the initiation of liver inflammation/ recruitment of leukocytes to the liver (83). An acute inflammatory response was clearly initiated in liver by YRF injection of trout, as shown by the up-regulation of many inflammatory genes. Interestingly, the major specific primary targets of TLR5 agonists (eg flagellin) in liver of rodents and primates are hepatocytes (84). The most inducible genes in trout liver were IL-11, IL-23P19 and IL-17C1 that were induced over 1,000 fold at 6 hpi, and remained elevated to 24 hpi. However, *in vitro* IL-11 was only moderately induced by YRF in the macrophage cell line RTS-11 and head kidney (HK) cells that contain macrophages (as well as other leucocytes), IL-23P19 was induced in RTS-11 but not HK cells and IL-17C1 was not induced in either RTS-11 or HK cells (23). This difference in immune gene induction may suggest different cells in liver are involved in the YRF response. Whether these cells are hepatocytes or other APCs remains to be determined in fish.

SAA (APP) and CATH2 (AMP) were massively induced by YRF in liver, especially at the late time point. Their later upregulation may suggest that these genes are affected by the cytokines (IL-11, IL-23, and IL-17C1) induced early by YRF, alone or in combination.

The best way for mass application of immunostimulants in aquaculture is their use as dietary additives (9). The activation of an inflammatory response and multiple defense pathways by YRF injection in liver suggests that flagellin could be delivered via the diet if formulated to protect its degradation in the gastric and intestinal tract of fish.

### The Middle D2/D3 Domain Is the Main Target of the Host Immune Response

A strong IgM response was induced after YRF immunization in rainbow trout. The antiserum could decrease the bioactivity of YRF, and our results showed that these antibodies reacted against the middle D2/D3 domain. However, an antibody response is not desirable when flagellin is used as an immunostimulant or vaccine adjuvant, since the antibodies may affect the flagellin stimulatory effect on repeat exposure. Although there is no direct experimental evidence, the binding of antibodies may block flagellin binding to its receptor, or opsonize the flagellin for its removal. A strong antibody response to flagellin may also adversely affect the specific response to the target antigen (s) when used as an vaccine adjuvant. To mitigate against these undesirable effects, a flagellin could be modified to remove the middle D2/D3 domain to eliminate or reduce the antibody response but retain the immunostimulatory effects (17, 20).

### Full-Length YRF Is Necessary for Activation of Inflammation: An Evasion Mechanism of the Pathogen?

The immunological impact of flagellin stimulation has driven several bacterial pathogens to evolve mechanisms to escape TLR5-mediated host defense. For example, Listeria shuts off flagellin expression at the host temperature of 37°C, and in the major food-borne pathogen *Campylobacter jejuni* and the gastric pathogen *Helicobacter pylori*, flagellin structural changes lead to the evasion of TLR5 activation (85). The activation of TLR5 requires a specific structural conformation in flagellin that is mainly defined in the D1 domain although other domains may contribute. Thus, flagellin that has been modified by removing the middle D2/D3 domain can retain its TLR5 activation capacity (20). It is also worth noting that different host species may have different structural requirements for flagellin to activate TLR5 signaling, as shown in mammals and birds (85). Our results showed that only full-length YRF is bioactive; any changes at the N-terminal, the C-terminal or the middle D2/D3 domain led to loss of bioactivity *in vitro*. This data suggests that TLR5 signaling in rainbow trout requires a structural conformation maintained by full-length YRF.

YRF originates from *Yersinia ruckeri*, a flagellated Gram-negative enterobacterium that is the causative agent of enteric redmouth disease (ERM), a hemorrhagic septicemia that primarily affects farmed salmonid fish species (23). A bacterin vaccine against ERM prepared from motile serovar 1 *Y. ruckeri* strains was the first commercialized fish vaccine. This vaccine has been used worldwide for several decades and has proven an effective and economical means for the control of ERM. It appears that the flagellum is necessary for vaccine-mediated immunity and that the prolonged use of this vaccine has provided a selective force driving the emergence of nonmotile variants associated with disease outbreaks in previously vaccinated fish, independently in the USA and Europe (86). These cases suggest that flagellin is an important virulence factor of motile *Y. ruckeri* and an important immune determinant of the host immune response and vaccine mediated protection. The requirement of a full-length YRF for TLR5 activation in rainbow trout may benefit this motile pathogen to evade TLR5 activation. It is perceivable that the required structural conformation might be easily disturbed by protease cleavage after flagellin release, or by binding of other proteins or antibodies *in vivo* or at the mucosal surface of the fish host.

In contrast to the single membrane form of TLR5 in mammals, teleost fish (including salmonids) possess one membrane TLR5 and one secreted TLR5. Both TLR5M and TLR5S bind flagellin (24). The modulation of TLR5M and TLR5S expression by flagellin is controversial in fish. In rainbow trout, YRF did not induce the expression of TLR5M and TLR5S *in vitro* in either RTS-11 cells or in primary HK cells (23) and only up-regulated the expression of TLR5S *in vivo*, as shown in this study. A strong up-regulation of TLR5S has also been reported in Atlantic salmon after vaccination with *V. anguillarum* flagellin (16). In channel catfish, both TLR5M and TLR5S are upregulated by recombinant *Y. ruckeri* flagellins *in vitro* in primary HK macrophages and *in vivo* in HK (87). It is noteworthy that fish TLR5M and TLR5S can be upregulated by bacterial infection and by LPS (24, 87). Clearly the fish species-specific modulation of TLR5 expression in response to flagellin needs further research in different species using pure flagellins.

In the present study rainbow trout TLR5S expression was induced rapidly *in vivo* in all the tissues examined at 6 hpi and this was sustained in the liver, gills and intestine to 24 hpi when the expression of most proinflammatory cytokines activated by flagellin had subsided. The different expression patterns suggest that trout TLR5S may function as a negative feedback regulator of flagellin signaling, that is induced by flagellin and competes with TLR5M for flagellin binding. This notion is in contrast to the hypothesis that the inducible TLR5S amplifies membrane TLR5-mediated cellular responses in a positive feedback fashion (24), and warrants further investigation to understand how flagellin signaling is regulated in fish.

### Using Flagellin as an Immunostimulant or Vaccine Adjuvant

Flagellin is a potent activator of innate immune responses *in vitro* in different fish species (23, 87). Our results in rainbow trout show that a transient systemic inflammation is activated following flagellin injection, leading to the activation of multiple antimicrobial pathways in both internal and mucosal tissues. Furthermore, non-specific protection against bacterial infections can be elicited in rainbow trout after flagellin injection (36). All these data suggest that flagellin can be used as a potent immunostimulant or a vaccine adjuvant in fish aquaculture.

The most convenient use of immunostimulants in aquaculture is as diet additives. Due to its protein nature, flagellin must be reformulated to avoid degradation in the gastric and intestinal tract, and should be modified by removing the antigenic D2/D3 domain. Although it is not possible to obtain functional mutants with YRF, research by others (85, 88) and by us suggests that some flagellins are malleable and functional mini-flagellins can be generated.

A vaccine-mediated immune response involves the integration of three distinct signals delivered in sequence. Signal 1 is antigen recognition; signal 2 is co-stimulation provided by APC; and signal 3 is the cytokine milieu that drives lymphocyte differentiation and expansion. Adjuvants can be distinguished between signal 1 facilitators and signal 2 facilitators. The signal 1 facilitators influence the fate of the vaccine antigen in time, place, and concentration, ultimately improving its immune-availability, while signal 2 facilitators provide the co-stimulatory signals during antigen recognition (8).

Flagellin can be a signal 1 facilitator as it can induce the expression of proinflammatory cytokines and chemokines eg CXCL8 and CXCL13, which promote the recruitment of T and B lymphocytes and APCs to the site of immunization, thus maximizing the chances of antigen-specific lymphocytes encountering their cognate antigens. It may also function as a signal 2 facilitator by activating APCs to express co-stimulatory receptors. Furthermore, flagellin in combination with other TLR agonists may function as a signal 3 facilitator by induction of cytokines for specific T helper cell subsets. For example, the induction of IL-4/13 expression by flagellin in rainbow trout may facilitate a Th2 type response, whilst IL-12 and IL-23 expression may drive Th1 and Th17 responses, respectively. It is widely accepted that as an adjuvant, flagellin can induce an enhanced antigen-specific immune response in different animals (31–35, 88).

In vaccine development for fish aquaculture, flagellin could be added to existing bacterin or inactivated viral vaccines by mixing or conjugating to enhance their immunogenicity. It could also be formulated in subunit vaccines as fusion proteins. The uniqueness of flagellin as an adjuvant is its ability to be easily incorporated into a DNA vaccine. DNA vaccines induce both early and late immune responses in fish and more than 20 different viral DNA vaccines have been developed experimentally for prophylactic use in fish. The rhabdoviridae DNA vaccines (eg VHSV and IHNV) have shown high levels of efficacy, athough with some viruses only moderate to low efficacies are seen (12). Thus, incorporation of adjuvants into these less effective vaccines may be needed to provide adequate protection. As a protein adjuvant, flagellin can be easily incorporated into a DNA vaccine as a DNA construct, or with the target antigen physically linked to the N- or C-terminal or replacing the D2/D3 domain. Thus, flagellin adjuvants have the potential to open new avenues for efficacious vaccine development for fish aquaculture.

## Conclusion

Of three bacterial PAMPs (LPS, peptidoglycan and flagellin) tested in this study, flagellin (YRF) was the most potent activator of proinflammatory cytokine expression *in vitro*. YRF injection of trout initiated a transient systemic inflammatory response with key pro-inflammatory cytokines (IL-1β, TNFα, IL-6, and IL-11 etc.) and chemokines (CXCL_F4 and CXCL-8), induced rapidly (6 hpi) but subsiding quickly in multiple tissues. Consequently, several anti-microbial pathways were activated systemically with heightened expression of APPs (SAA), AMPs (cathelicidins and hepcidin) and complement genes in multiple tissues, which was sustained to 24 hpi in the liver and mucosal tissues. The Th17 cytokines IL-17A/F1a and IL-17A/F2a were also induced in the liver and spleen (IL-17A/F1a only), and Th2 cytokine IL-4/13 was induced in the liver. However, the anti-inflammatory IL-10 and Th1 cytokine IFNγ were refractory. The secreted TLR5S was induced by flagellin in all tissues examined whilst the membrane TLR5M was refractory, suggesting that TLR5S may function as a negative feedback regulator. Trout liver appeared to be an important organ responding to flagellin stimulation, with marked induction of IL-11, IL-23P19, IL-17C1, SAA and cathelicidin-2. YRF induced a strong antibody response, with the generated antibodies binding the middle domain of YRF and able to neutralize YRF bioactivity. Intact YRF is necessary for its bioactivity, with deletion of either the N-terminal, C terminal or middle domain of YRF leading to functional loss. This study adds to the growing literature suggesting that flagellin could be a potent immunostimulant and vaccine adjuvant for fish aquaculture.

## Ethics Statement

All the experiments described comply with the Guidelines of the European Union Council (2010/63/EU) for the use of laboratory animals, and were carried out under UK Home Office project license PPL 60/4013, approved by the ethics committee at the University of Aberdeen.

## Author Contributions

TW conceived and planned the study, analyzed and interpreted the data, and wrote the first draft of the manuscript. EW performed experiments, analyzed the data, and wrote the paper. CS conceived and planned the study, analyzed and interpreted the data, and wrote the paper. All authors read and approved the final manuscript.

### Conflict of Interest Statement

The authors declare that the research was conducted in the absence of any commercial or financial relationships that could be construed as a potential conflict of interest.

## References

[1] FAO The State of World Fisheries and Aquaculture 2016: Contributing to food security and nutrition for all. Rome: FAO p. 200 (2016). Available online at: http://www.fao.org/3/a-i5555e.pdf

[2] Kibenge FS Godoy MG Fast M Workenhe S Kibenge MJ. Countermeasures against viral diseases of farmed fish. Antiviral Res. (2012) 95:257–81. 10.1016/j.antiviral.2012.06.003 22721634

[3] Lafferty KD Harvell CD Conrad JM Friedman CS Kent ML Kuris AM. Infectious diseases affect marine fisheries and aquaculture economics. Ann Rev Mar Sci. (2015) 7:471–96. 10.1146/annurev-marine-010814-015646 25251276

[4] Santos L Ramos F. Antimicrobial resistance in aquaculture: current knowledge and alternatives to tackle the problem. Int J Antimicrob Agents (2018) 52:135–143. 10.1016/j.ijantimicag.2018.03.010 29567094

[5] Assefa A Abunna F. Maintenance of fish health in aquaculture: review of epidemiological approaches for prevention and control of infectious disease of fish. Vet Med Int. (2018) 2018:5432497. 10.1155/2018/5432497 29682272 PMC5846361

[6] Pérez-Sánchez T Mora-Sánchez B Balcázar JL. Biological approaches for disease control in aquaculture: advantages, limitations and challenges. Trends Microbiol. (2018) 26:896–903. 10.1016/j.tim.2018.05.002 29801773

[7] Newaj-Fyzul A Austin B. Probiotics, immunostimulants, plant products and oral vaccines, and their role as feed supplements in the control of bacterial fish diseases. J Fish Dis. (2015) 38:937–55. 10.1111/jfd.12313 25287254

[8] Tafalla C Bøgwald J Dalmo RA. Adjuvants and immunostimulants in fish vaccines: current knowledge and future perspectives. Fish Shellfish Immunol. (2013) 35:1740–50. 10.1016/j.fsi.2013.02.029 23507338

[9] Vallejos-Vidal E Reyes-López F Teles M MacKenzie S. The response of fish to immunostimulant diets. Fish Shellfish Immunol. (2016) 56:34–69. 10.1016/j.fsi.2016.06.028 27389620

[10] Librán-Pérez M Costa MM Figueras A Novoa B. β-glucan administration induces metabolic changes and differential survival rates after bacterial or viral infection in turbot (*Scophthalmus maximus*). Fish Shellfish Immunol. (2018) 82:173–182. 10.1016/j.fsi.2018.08.005 30081180

[11] Munang'andu HM. Intracellular bacterial infections: a challenge for developing cellular mediated immunity vaccines for farmed fish. Microorganisms (2018) 6:E33. 10.3390/microorganisms6020033 29690563 PMC6027125

[12] Dalmo RA. DNA vaccines for fish: review and perspectives on correlates of protection. J Fish Dis. (2018) 41:1–9. 10.1111/jfd.12727 29064091

[13] Del Giudice G Rappuoli R Didierlaurent AM. Correlates of adjuvanticity: a review on adjuvants in licensed vaccines. Semin Immunol. (2018) 39:14–21. 10.1016/j.smim.2018.05.001 29801750

[14] Chettri JK Raida MK Holten-Andersen L Kania PW Buchmann K. PAMP induced expression of immune relevant genes in head kidney leukocytes of rainbow trout (*Oncorhynchus mykiss*). Dev Comp Immunol. (2011) 35:476–82. 10.1016/j.dci.2010.12.001 21147161

[15] Kawai T Akira S. The role of pattern-recognition receptors in innate immunity: update on Toll-like receptors. Nature Immunol. (2010) 11:373–84. 10.1038/ni.1863 20404851

[16] Hynes NA Furnes C Fredriksen BN Winther T Bøgwald J Larsen AN Dalmo RA. Immune response of Atlantic salmon to recombinant flagellin. Vaccine (2011) 29:7678–87. 10.1016/j.vaccine.2011.07.138 21843579

[17] Yoon SI Kurnasov O Natarajan V Hong M Gudkov AV Osterman AL Wilson IA. Structural basis of TLR5-flagellin recognition and signalling. Science (2012) 335:859–64. 10.1126/science.1215584 22344444 PMC3406927

[18] Akira S Uematsu S Takeuchi O. Pathogen recognition and innate immunity. Cell (2006) 124:783–801. 10.1016/j.cell.2006.02.015 16497588

[19] Zhao Y Shao F. The NAIP-NLRC4 inflammasome in innate immune detection of bacterial flagellin and type III secretion apparatus. Immunol Rev. (2015) 265:85–102. 10.1111/imr.12293 25879286

[20] Il Kim M Lee C Park J Jeon BY Hong M. Crystal structure of *Bacillus cereus* flagellin and structure-guided fusion-protein designs. Sci Rep. (2018) 8:5814. 10.1038/s41598-018-24254-w 29643437 PMC5895748

[21] Hayashi F Smith KD Ozinsky A Hawn TR Yi EC Goodlett DR. The innate immune response to bacterial flagellin is mediated by Toll-like receptor 5. Nature (2001) 410:1099–103. 10.1038/35074106 11323673

[22] Okugawa S Yanagimoto S Tsukada K Kitazawa T Koike K Kimura S. Bacterial flagellin inhibits T cell receptor-mediated activation of T cells by inducing suppressor of cytokine signalling-1 (SOCS-1). Cell Microbiol. (2006) 8:1571–80. 10.1111/j.1462-5822.2006.00731.x 16984412

[23] Wangkahart E Scott C Secombes CJ Wang T. Re-examination of the rainbow trout (*Oncorhynchus mykiss*) immune response to flagellin: *Yersinia ruckeri* flagellin is a potent activator of acute phase proteins, anti-microbial peptides and pro-inflammatory cytokines *in vitro*. Dev Comp Immunol. (2016) 57:75–87. 10.1016/j.dci.2015.12.017 26719024

[24] Tsujita T Tsukada H Nakao M Oshiumi H Matsumoto M Seya T. Sensing bacterial flagellin by membrane and soluble orthologs of Toll-like receptor 5 in rainbow trout (*Oncorhynchus mykiss*). J Biol Chem. (2004) 279:48588–97. 10.1074/jbc.M407634200 15339910

[25] Tsoi S Park KC Kay HH O'Brien TJ Podor E Sun G. Identification of a transcript encoding a soluble form of toll-like receptor 5 (TLR5) in Atlantic salmon during *Aeromonas salmonicida* infection. Vet Immunol Immunopathol. (2006) 109:183–7. 10.1016/j.vetimm.2005.05.013 16112748

[26] Baoprasertkul P Xu P Peatman E Kucuktas H Liu Z. Divergent Toll-like receptors in catfish (*Ictalurus punctatus*): TLR5S, TLR20, TLR21. Fish Shellfish Immunol. (2007) 23:1218–30. 10.1016/j.fsi.2007.06.002 17981052

[27] Hwang SD Asahi T Kondo H Hirono I Aoki T. Molecular cloning and expression study on Toll-like receptor 5 paralogs in Japanese flounder, *Paralichthys olivaceus*. Fish Shellfish Immunol. (2010) 29:630–8. 10.1016/j.fsi.2010.06.011 20561590

[28] Muñoz I Sepulcre MP Meseguer J Mulero V. Molecular cloning, phylogenetic analysis and functional characterization of soluble Toll-like receptor 5 in gilthead seabream, *Sparus aurata*. Fish Shellfish Immunol. (2013) 35:36–4. 10.1016/j.fsi.2013.03.374 23571319

[29] Bai JS Li YW Deng Y Huang YQ He SH Dai J. Molecular identification and expression analysis of TLR5M and TLR5S from orange-spotted grouper (*Epinepheluscoioides*). Fish Shellfish Immunol. (2017) 63:97–102. 10.1016/j.fsi.2017.01.037 28159696

[30] Zhang M Wu H Li X Yang M Chen T Wang Q. *Edwardsiella tarda* flagellar protein FlgD: a protective immunogen against *edwardsiellosis*. Vaccine (2012) 30:3849–56. 10.1016/j.vaccine.2012.04.008 22521284

[31] Jia PP Hu YH Chi H Sun BG Yu WG Sun L. Comparative study of four flagellins of *Vibrio anguillarum*: vaccine potential and adjuvanticity. Fish Shellfish Immunol. (2013) 34:514–20. 10.1016/j.fsi.2012.11.039 23253494

[32] Liu X Zhang H Jiao C Liu Q Zhang Y Xiao J. Flagellin enhances the immunoprotection of formalin-inactivated *Edwardsiella tarda* vaccine in turbot. Vaccine (2017) 35:369–74. 10.1016/j.vaccine.2016.11.031 27908640

[33] Xing J Zhou X Tang X Sheng X Zhan W. FlaC supplemented with VAA, OmpK or OmpR as bivalent subunit vaccine candidates induce immune responses against *Vibrio anguillarum* in flounder (*Paralichthys olivaceus*). Vaccine (2018) 36:1316–22 10.1016/j.vaccine.2017.11.074 29397223

[34] Beck BR Lee SH Kim D Park JH Lee HK Kwon SS. A Lactococcus lactis BFE920 feed vaccine expressing a fusion protein composed of the OmpA and FlgD antigens from *Edwardsiella tarda* was significantly better at protecting olive flounder (*Paralichthys olivaceus*) from *edwardsiellosis* than single antigen vaccines. Fish Shellfish Immunol. (2017) 68:19–28. 10.1016/j.fsi.2017.07.004 28687358

[35] Liang HY Wu ZH Jian JC Liu ZH. Construction of a fusion flagellin complex and evaluation of the protective immunity of it in red snapper (*Lutjanus sanguineus*). Lett Appl Microbiol. (2012) 55:115–21. 10.1111/j.1472-765X.2012.03267.x 22591449

[36] Scott CJW Austin B Austin DA Morris PC. Non-adjuvanted flagellin elicits a non-specific protective immune response in rainbow trout (*Oncorhynchus mykiss*, Walbaum) towards bacterial infections. Vaccine (2013) 31:3262–7. 10.1016/j.vaccine.2013.05.025 23707165

[37] González-Stegmaie R Romero A Estepa A Montero J Mulero V Mercado L. Effects of recombinant flagellin B and its ND1 domain from *Vibrio anguillarum* on macrophages from gilthead seabream (*Sparus aurata* L.) and rainbow trout (*Oncorhynchus mykiss*, W.). Fish Shellfish Immunol. (2015) 42:144–52. 10.1016/j.fsi.2014.10.034 25449380

[38] Cesta MF. Normal structure, function, and histology of the spleen. Toxicol Pathol. (2006) 34:455–65. 10.1080/01926230600867743 17067939

[39] Awasthi A Rathore G Pradhan PK Rebello SC Khan MY Lakra WS. Isolation and characterization of head kidney derived macrophages of *Labeo rohita*. J Environ Biol. (2014) 35:949. 25204072

[40] Rombout JHWM Taverne-Thiele AJ Villena MI. The gut-associated lymphoid tissue (GALT) of carp (*Cyprinus carpio* L.): an immunocytochemical analysis. Dev Comp Immunol. (1993) 17:55–66. 10.1016/0145-305X(93)90015-I 8449251

[41] Xu Z Parra D Gómez D Salinas I Zhang YA Jørgensen L von G. Teleost skin, an ancient mucosal surface that elicits gut-like immune response. Proc Natl Acad Sci USA. (2013) 110:13097–102. 10.1073/pnas.1304319110 23884653 PMC3740891

[42] Salinas I Magadán S. Omics in fish mucosal immunity. Dev Comp Immunol. (2017) 75:99–108. 10.1016/j.dci.2017.02.010 28235585

[43] Wangkahart E Secombes CJ Wang T. Dissecting the immune pathways stimulated following injection vaccination of rainbow trout (*Oncorhynchus mykiss*) against enteric redmouth disease (ERM). Fish Shellfish Immunol. (2017) 10.1016/j.fsi.2017.07.056. [Epub ahead of print]. 28757198

[44] Wang T Husain M Hong S Holland JW. Differential expression, modulation and bioactivity of distinct fish IL-12 isoforms: implication towards the evolution of Th1-like immune responses. Eur J Immunol. (2014) 44:1541–51. 10.1002/eji.201344273 24470165

[45] Ganassin RC Bols NC Development of a monocyte/macrophage-like cell line, RTS11, from rainbow trout spleen. Fish Shellfish Immunol. (1998) 8:457–76. 10.1006/fsim.1998.0153

[46] Wang T Husain M. The expanding repertoire of the IL-12 cytokine family in teleost fish: identification of three paralogues each of the p35 and p40 genes in salmonids, and comparative analysis of their expression and modulation in Atlantic salmon *Salmo salar*. Dev Comp Immunol. (2014) 46:194–207. 10.1016/j.dci.2014.04.008 24759618

[47] Wang T Diaz-Rosales P Costa MM Campbell S Snow M Collet B. Functional characterization of a nonmammalian IL-21: rainbow trout *Oncorhynchus mykiss* IL-21 upregulates the expression of the Th cell signature cytokines IFN-gamma, IL-10, and IL-22. J Immunol. (2011) 186:708–21. 10.4049/jimmunol.1001203 21160047

[48] Wang T Huang W Costa MM Martin SA Secombes CJ. Two copies of the genes encoding the subunits of putative interleukin (IL)-4/IL-13 receptors, IL-4Rα, IL-13Rα1 and IL-13Rα2, have been identified in rainbow trout (*Oncorhynchus mykiss*) and have complex patterns of expression and modulation. Immunogenetics (2011) 63:235–53. 10.1007/s00251-010-0508-2 21210100

[49] DeLuca D Wilson M Warr GW. Lymphocyte heterogeneity in the trout, *Salmo gairdneri*, defined with monoclonal antibodies to IgM. Eur J Immunol. (1983) 13:546–51. 10.1002/eji.1830130706 6347695

[50] Husain M Bird S van Zwieten R Secombes CJ Wang T. Cloning of the IL-1β3 gene and IL-1β4 pseudogene in salmonids uncovers a second type of IL-1β gene in teleost fish. Dev Comp Immunol. (2012) 38:431–46. 10.1016/j.dci.2012.07.010 22889890

[51] Hong S Li R Xu Q Secombes CJ Wang T. Two types of TNF-α exist in teleost fish: phylogeny, expression, and bioactivity analysis of type-II TNF-α3 in rainbow trout *Oncorhynchus mykiss*. J Immunol. (2013) 191:5959–72. 10.4049/jimmunol.1301584 24244011

[52] Costa MM Maehr T Diaz-Rosales P Secombes CJ Wang T. Bioactivity studies of rainbow trout (*Oncorhynchus mykiss*) interleukin-6: effects on macrophage growth and antimicrobial peptide gene expression. Mol Immunol. (2011) 48:1903–16. 10.1016/j.molimm.2011.05.027 21704380

[53] Zou J Bird S Truckle J Bols N Horne M Secombes CJ. Identification and expression analysis of an IL-18 homologue and its alternatively spliced form in rainbow trout (*Oncorhynchus mykiss*). Eur J Biochem. (2004) 271:1913–23. 10.1111/j.1432-1033.2004.04101.x 15128301

[54] Wang T Bird S Koussounadis A Holland JW Carrington A Zou J. Identification of a novel IL-1 cytokine family member in teleost fish. J Immunol. (2009) 183:962–74. 10.4049/jimmunol.0802953 19553537

[55] Wang T Holland JW Bols N Secombes CJ. Cloning and expression of the first nonmammalian interleukin-11 gene in rainbow trout *Oncorhynchus mykiss*. FEBS J. (2005) 272:1136–47. 10.1111/j.1742-4658.2005.04544.x 15720388

[56] Wang T Secombes CJ. Identification and expression analysis of two fish-specific IL-6 cytokine family members, the ciliary neurotrophic factor (CNTF)-like and M17 genes, in rainbow trout *Oncorhynchus mykiss*. Mol Immunol. (2009) 46:2290–8. 10.1016/j.molimm.2009.04.003 19419770

[57] Wang T Kono T Monte MM Kuse H Costa MM Korenaga H. Identification of IL-34 in teleost fish: differential expression of rainbow trout IL-34, MCSF1 and MCSF2, ligands of the MCSF receptor. Mol Immunol. (2013) 53:398–409. 10.1016/j.molimm.2012.09.008 23099477

[58] Chen J Xu Q Wang T Collet B Corripio-Miyar Y Bird S. Phylogenetic analysis of vertebrate CXC chemokines reveals novel lineage specific groups in teleost fish. Dev Comp Immunol. (2013) 41:137–52. 10.1016/j.dci.2013.05.006 23701879

[59] Jiang Y Husain M Qi Z Bird S Wang T. Identification and expression analysis of two interleukin-23α (p19) isoforms, in rainbow trout *Oncorhynchus mykiss* and Atlantic salmon Salmo salar. Mol Immunol. (2015) 66:216–28. 10.1016/j.molimm.2015.03.014 25841173

[60] Husain M Martin SAM Wang T. Identification and characterisation of the IL-27 p28 subunits in fish: cloning and comparative exression analysis of two p28 paralogues in Atlantic salmon *Salmo salar*. Fish Shellfish Immunol. (2014) 41:102–12. 10.1016/j.fsi.2014.06.024 24981291

[61] Secombes CJ Wang T Bird S. The interleukins of fish. Dev Comp Immunol. (2011) 35:1336–45. 10.1016/j.dci.2011.05.001 21605591

[62] Wang T Díaz-Rosales P Martin SA Secombes CJ. Cloning of a novel interleukin (IL)-20-like gene in rainbow trout *Oncorhynchus mykiss* gives an insight into the evolution of the IL-10 family. Dev Comp Immunol. (2010) 34:158–67. 10.1016/j.dci.2009.09.003 19755128

[63] Wang T Jiang Y Wang A Husain M Xu Q Secombes CJ. Identification of the salmonid IL-17A/F1a/b, IL-17A/F2b, IL-17A/F3 and IL-17N genes and analysis of their expression following in vitro stimulation and infection. Immunogenetics (2015) 67:395–412. 10.1007/s00251-015-0838-1 25943775

[64] Monte MM Wang T Holland JW Zou J Secombes CJ. Cloning and characterization of rainbow trout interleukin-17A/F2 (IL-17A/F2) and IL-17 receptor A: expression during infection and bioactivity of recombinant IL-17A/F2. Infect Immun. (2013) 81:340–53. 10.1128/IAI.00599-12 23147036 PMC3536140

[65] Wang T Martin SAM Secombes CJ. Two interleukin-17C-like genes exist in rainbow trout *Oncorhynchus mykiss* that are differentially expressed and modulated. Dev Comp Immunol. (2010) 34:491–500. 10.1016/j.dci.2009.11.011 19961871

[66] Monte MM Zou J Wang T Carrington A Secombes CJ. Cloning, expression analysis and bioactivity studies of rainbow trout (*Oncorhynchus mykiss*) interleukin-22. Cytokine (2011) 55:62–73. 10.1016/j.cyto.2011.03.015 21514178

[67] Wang T Johansson P Abós B Holt A Tafalla C Jiang Y. First in-depth analysis of the novel Th2-type cytokines in salmonid fish reveals distinct patterns of expression and modulation but overlapping bioactivities. Oncotarget (2016) 7:10917–46. 10.18632/oncotarget.7295 26870894 PMC4905449

[68] Wang T Holland JW Carrington A Zou J Secombes CJ. Molecular and functional characterization of IL-15 in rainbow trout *Oncorhynchus mykiss*: a potent inducer of IFN-γ expression in spleen leukocytes. J Immunol. (2007) 179:1475–88. 10.4049/jimmunol.179.3.1475 17641013

[69] Wang T Hu Y Wangkahart E Liu F Wang A Zahran E. Interleukin (IL)-2 Is a Key Regulator of T Helper 1 and T Helper 2 Cytokine Expression in Fish: functional characterization of two divergent IL2 paralogs in salmonids. Front Immunol. (2018) 9:1683. 10.3389/fimmu.2018.01683. 30093902 PMC6070626

[70] Harun NO Costa MM Secombes CJ Wang T. Sequencing of a second interleukin-10 gene in rainbow trout *Oncorhynchus mykiss* and comparative investigation of the expression and modulation of the paralogues *in vitro* and *in vivo*. Fish Shellfish Immunol. (2011) 31:107–17. 10.1016/j.fsi.2011.04.010 21536138

[71] Maehr T Costa MM Vecino JLG Wadsworth S Martin SAM Wang T Secombes CJ. Transforming growth factor-β1b: a second TGF-β1 paralogue in the rainbow trout (*Oncorhynchus mykiss*) that has a lower constitutive expression but is more responsive to immune stimulation. Fish Shellfish Immunol. (2013) 34:420–32. 10.1016/j.fsi.2012.11.011 23178261

[72] Wang B Wangkahart E Secombes CJ Wang T. Insights into the evolution of the suppressors of cytokine signalling (SOCS) gene family in vertebrates. Mol Biol Evol. (2018) 10.1093/molbev/msy230. [Epub ahead of print]. 30521052 PMC6368001

[73] Maehr T Vecino JL Wadsworth S Wang T Secombes CJ. Four CISH paralogues are present in rainbow trout *Oncorhynchus mykiss*: differential expression and modulation during immune responses and development. Mol Immunol. (2014) 62:186–98. 10.1016/j.molimm.2014.06.021 25014904

[74] Villarroel F Casado A Vásquez J Matamala E Araneda B Amthauer R. Serum amyloid A: a typical acute-phase reactant in rainbow trout? Dev Comp Immunol. (2008) 32:1160–9. 10.1016/j.dci.2008.03.004 18440634

[75] Chang CI Zhang YA Zou J Nie P Secombes. Two cathelicidin genes are present in both rainbow trout (*Oncorhynchus mykiss*) and Atlantic salmon (*Salmo salar*) Antimicrob. Antimicrob Agents Chemother. (2006) 50:185–95. 10.1128/AAC.50.1.185-195.2006 16377685 PMC1346769

[76] Douglas SE Gallant JW Liebscher RS Dacanay A Tsoi SC. Identification and expression analysis of hepcidin-like antimicrobial peptides in bony fish. Dev Comp Immunol. (2003) 27:589–601. 10.1016/S0145-305X(03)00036-3 12697315

[77] Casadei E Wang T Zou J González Vecino JL Wadsworth S Secombes CJ. Characterization of three novel beta-defensin antimicrobial peptides in rainbow trout (*Oncorhynchus mykiss*). Mol Immunol. (2009) 46:3358–66. 10.1016/j.molimm.2009.07.018 19709750

[78] Köbis JM Rebl A Kühn C Korytár T Köllner B Goldammer T. Comprehensive and comparative transcription analyses of the complement pathway in rainbow trout. Fish Shellfish Immunol. (2015) 42:98–107. 10.1016/j.fsi.2014.10.032 25449374

[79] Grayfer L Kerimoglu B Yaparla A Hodgkinson JW Xie J Belosevic M. Mechanisms of fish macrophage antimicrobial immunity. Front Immunol. (2018) 9:1105. 10.3389/fimmu.2018.01105 29892285 PMC5985312

[80] Edholm ES Rhoo KH Robert J. Evolutionary aspects of macrophages polarization. Results Probl Cell Differ. (2017) 62:3–22. 10.1007/978-3-319-54090-0_1 28455703 PMC5695037

[81] Benedicenti O Wang T Wangkahart E Milne DJ Holland JW Collins C Secombes CJ. Characterisation of arginase paralogues in salmonids and their modulation by immune stimulation/infection. Fish Shellfish Immunol. (2017) 61:138–151. 10.1016/j.fsi.2016.12.024 28025160

[82] Wang T Secombes CJ. The cytokine networks of adaptive immunity in fish. Fish Shellfish Immunol. (2013) 35:1703–18. 10.1016/j.fsi.2013.08.030 24036335

[83] Carambia A Herkel J. Dietary and metabolic modulators of hepatic immunity. Semin Immunopathol. (2018) 40:175–88. 10.1007/s00281-017-0659-4 29110070

[84] Burdelya LG Brackett CM Kojouharov B Gitlin II Leonova KI Gleiberman AS Aygun-Sunar S. Central role of liver in anticancer and radioprotective activities of Toll-like receptor 5 agonist. Proc Natl Acad Sci USA. (2013) 110:E1857–66. 10.1073/pnas.1222805110 23630282 PMC3657788

[85] de Zoete MR Keestra AM Wagenaar JA van Putten JP. Reconstitution of a functional Toll-like receptor 5 binding site in *Campylobacter jejuni* flagellin. J Biol Chem. (2010) 285:12149–58 10.1074/jbc.M109.070227 20164175 PMC2852954

[86] Welch TJ Verner-Jeffreys DW Dalsgaard I Wiklund T Evenhuis JP Garcia JA. Independent emergence of *Yersinia ruckeri* biotype 2 in the United States and Europe. Appl Env Micriobiol. (2011) 77:3493–9. 10.1128/AEM.02997-10 21441334 PMC3126439

[87] Jiang J Zhao W Xiong Q Wang K He Y Wang J. Immune responses of channel catfish following the stimulation of three recombinant flagellins of *Yersinia ruckeri in vitro* and *in vivo*. Dev Comp Immunol. (2017) 73:61–71. 10.1016/j.dci.2017.02.015 28235583

[88] Cui B Liu X Fang Y Zhou P Zhang Y Wang Y. Flagellin as a vaccine adjuvant. Expert Rev Vaccines (2018) 17:335–49. 10.1080/14760584.2018.1457443 29580106

