# Supplementary material for: Studies on the Use of Flagellin as an Immunostimulant and Vaccine Adjuvant in Fish Aquaculture
Source: Front Immunol. 2019 Jan 9;9:3054. doi: 10.3389/fimmu.2018.03054 (PMC6333709; doi:10.3389/fimmu.2018.03054)
Supplement: Supplementary file 1 [file Data_Sheet_1.pdf]

Supplementary materials:

Studies on the use of flagellin as an immunostimulant and vaccine adjuvant in fish aquaculture

Eakapol Wangkahart, Christopher J. Secombes and Tiehui Wang

Figure S1 A multiple alignment of recombinant *Y. ruckeri* flagellin (YRF) and its muteins YRF-N, YRF-C and YRF-NC.

Table S1 Primers used for real-time PCR analysis of gene expression

Table S2 Fold change of transcript expression of immune-relevant genes in the spleen, liver, gills and skin of fish ip injected with YRF.

```
YRF             MGAVINTNSLSLLTQNNLNKSQGSLGTAIERLSSGLRINSAKDDAAGQAIANRFTSNING 60
YRF-NC          MGAVINTNSLSLLTQNNLNKSQGSLGTAIERLSSGLRINSAKDDAAGQAIANRFTSNING 60
YRF-N           MGAVINTNSLSLLTQNNLNKSQGSLGTAIERLSSGLRINSAKDDAAGQAIANRFTSNING 60
YRF-C           ------------------------------------------------------------ 0


YRF             LTQAARNANDGISLSQTAEGALGEINNNLQRVRDLTVQAQNSSNSASDIDSIQSEVNQRM 120
YRF-NC          LTQAARNANDGISLSQTAEGALGEINNNLQRVRDLTVQAQNSSNSASDIDSIQSEVNQRM 120
YRF-N           LTQAARNANDGISLSQTAEGALGEINNNLQRVRDLTVQAQNSSNSASDIDSIQSEVNQRM 120
YRF-C           ------------------------------------------------------------ 0


YRF             EEINRVTKQTDFNGIKVLDNRTAANAEYAFQVGSQDAQKINIEIGSSAGWNLATAGAGGT 180
YRF-NC          EEINRVTKQTDFNGIKVLSGGGGSGGGGSGGGGS-------------------------- 148
YRF-N           EEINRVTKQTDFNGIKVLDNRTAANAEYAFQVGSQDAQKINIEIGSSAGWNLATAGAGGT 180
YRF-C           ----------------MGDNRTAANAEYAFQVGSQDAQKINIEIGSSAGWNLATAGAGGT 44
                                :

YRF             SSDVVNDSTQISKAKETVVQTLSGKTEAQINTALTKFTTDVKAATDAAGVVTAKGALTTA 240
YRF-NC          ------------------------------------------------------------ 148
YRF-N           SSDVVNDSTQISKAKETVVQTLSGKTEAQINTALTKFTTDVKAATDAAGVVTAKGALTTA 240
YRF-C           SSDVVNDSTQISKAKETVVQTLSGKTEAQINTALTKFTTDVKAATDAAGVVTAKGALTTA 104


YRF             LGLKADADLGTAVSSAAFGTDLSVDQIAGVKSGVYSAAINGANYATAKTEAEVSAAQAGA 300
YRF-NC          ------------------------------------------------------------ 148
YRF-N           LGLKADADLGTAVSSAAFGTDLSVDQIAGVKSGVYSAAINGANYATAKTEAEVSAAQAGA 300
YRF-C           LGLKADADLGTAVSSAAFGTDLSVDQIAGVKSGVYSAAINGANYATAKTEAEVSAAQAGA 164


YRF             KTAGAMVNGNFRSVEAKGFDVLKGNVTGGATGTATGTTPLADIDAALKAVDSQRSSLGAS 360
YRF-NC          -------------VEAKGFDVLKGNVTGGATGTATGTTPLADIDAALKAVDSQRSSLGAS 201
YRF-N           KTAGAMVNGNFRS----------------------------------------------- 327
YRF-C           KTAGAMVNGNFRSVEAKGFDVLKGNVTGGATGTATGTTPLADIDAALKAVDSQRSSLGAS 224


YRF             QNRFESTITNLNNTVNNLTSARSRIQDADYSTEVSNMSRAQILQQAGTSVLAQANQVPQT 420
YRF-NC          QNRFESTITNLNNTVNNLTSARSRIQDADYSTEVSNMSRAQILQQAGTSVLAQANQVPQT 261
YRF-N           ------------------------------------------------------------ 327
YRF-C           QNRFESTITNLNNTVNNLTSARSRIQDADYSTEVSNMSRAQILQQAGTSVLAQANQVPQT 284


YRF             VLSLLRASSAHHHHHHHHHH    440
YRF-NC          VLSLLRASSAHHHHHHHHHH    281
YRF-N           ------ASSAHHHHHHHHHH    327
YRF-C           VLSLLRASSAHHHHHHHHHH    304
```

Figure S1 A multiple alignment of recombinant *Y. ruckeri* flagellin (YRF) and its muteins YRF-N, YRF-C and YRF-NC. The aa sequences derived from the expression vector are highlighted in green. A GS linker (SGGGGSGGGGSGGGGS) inserted in YRF-NC is highlighted in red.

Table S1 Primers used for real-time PCR analysis of gene expression

Gene	Forward (5' to 3')	Reverse (5' to 3')	Acc. No.
**House-keeping gene**			
**EF-1α**	CAAGGATATCCGTCGTGGCA	ACAGCGAAACGACCAAGAGG	AF498320
**IL-1 Family**			
**IL-1β1**	CCTGGAGCATCATGGCGTG	GCTGGAGAGTGCTGTGGAAGAACATATAG	AJ278242
**IL-1β2**	GAGCGCAGTGGAAGTGTTGG	AGACAGGTTCAAATGCACTTTATGGT	AJ245925
**IL-1β3**	CTGAAGGCCGTCACAATCCA	CTGGTCCTTACAGCGCTCCAA	AM181685
**nIL-1Fm**	CCCATTCCTCGTGACACCAG	CTGGACGACCTGGAGAGTGACT	AJ555869
**IL-18**	GAGCAATGCAAAGCAGATGATTG	CATGTTTTGAGCAGCCAATGTAGTC	AJ556990
**TNF family**			
**TNFα1**	TGTGTGGGGTCCTCTTAATAGCAGGTC	CCTCAATTTCATCCTGCATCGTTGA	AJ277604
**TNFα2**	CTGTGTGGCGTTCTCTTAATAGCAGCTT	CATTCCGTCCTGCATCGTTGC	AJ401377
**TNFα3**	GCTGCACTCTTCTTTACCAAGAAACAAG	CCACTGAGGACTTGTAATCACCATAGGT	HE798544
**IL-6 family**			
**IL-6**	GGGAGAAAATGATCAAGATGCTCGT	GCAGACATGCCTCCTTGTTGG	DQ866150
**IL-11**	CTCTCGCTGCTATTGGCCCA	TCTCGAATGCATGTTCCTTCAATAGAT	AJ535687
**M17**	GTGGACCTCTTAAAAACATACAAGCTCAG	GGATGGTGGCTGTAAGTCTGTCTG	FM866399
**CNTF**	GCACTTATCTTCTGGAGCTATATAGGGAGA	AACTCCATCAACCTCCTCATTGC	FM866401
**IL-12 family**			
**P19**	ACCTAAGAGCAGATTCAATGCCTTG	TCTTCCCAGCTCTTCACTTCCTG	KP410548
**P28A**	GCAGCTGCTCAGGAGATATAAGGAGG	TCTCTCAGGTATGCTGGGTTTTGG	HG794528
**P28B**	GCAGCTGCTCATGAGATATAAAGAGGA	GCTGCTCTCTGTTCCACCTTATCCAC	HG794529
**P35A1**	GGAACACCACATTCAGTGAGAGTGC	CGTCTGCAACTTGTGAGGAAGGAT	HE798148
**P35A2**	GGAACACCACATTCAGTGAGAGTGA	CAACCTGTGAGGAAGACACCCA	HG917950
**P35B1**	TGCCAAACGCCAAGCTTTATTTTG	GCTGTTGAGTGCTTTTGGTCTTTGG	HG917951
**P40B1**	CCCTTCTACATCCGAGAAATAGTGAAAC	GTTGGTTTCACTTATAAACACCTTTTCCTT	HE798149
**P40B2**	CCGTTCTACATACGAGAAATAGTGGAGA	TCAGAGTCACAGCTTTCCCTGG	HG917952
**P40C**	TTAAAGACAACGGAAAGGAGGAGC	CCTCCCGTAACCACATTTTTCC	AJ548830
**EBI3**	ACATCGCCACCTACAGTATGAAAGG	GGGTCCGGCTTCACAATGT	AJ620467
**Chemokines**			
**CXCL_F1a**	TCATCTACATGATACAAGGG A	TCAAGTGCTAGGGGTGGTATG	DQ191448
**CXCL_F1b**	CGTCATCTGCATGATACAAGGGAGAAC	TCCGGATTTCAGTGTGACAATGAG	NM_001124562
**CXCL_F1c**	GCTGCCTTTGCCCCAAAGTG	CCTGCATTCTTTTGGTTGATC	HE578132
**CXCL_F2**	TCTGTGAGCCAACGATGTTTGTG	GCACTCCATTCTTAAGGCTAACAATGAA	BX859166
**CXCL_F4**	TGCCTACTCCTTACAGTGGGTA	TCCGCTGGTTTTCTTCCA	HE578134
**CXCL_F5**	TGGGAGTTTGTATACAGCTCAAC	CTTTCTTCCAACGGGGTTCAGG	HE578133
**CXCL8**	AGAGACACTGAGATCATTGCCAC	CCCTCTTCATTTGTTGTTGGC	AJ310565
**CXCL11_L1**	TCTGCTCCTGGCCAATGTTGAAG	TCCCTTCTCCGTTCTTCAGAGT	AJ417078
**CXCL12a**	GACATACGTTCCTTTCGCACA	GCTTTCTTCATCTTGTTGAGGGCA	HE578135
**CXCL12b**	CATGGATGTACCTCCCTCTACG	CTTCTTCACCTTGTTAATGGCA	HE578136
**CXCL13**	CAGGTTTCACAAGTTGGAGA	TCCCTTGTTGTTACTTTGTAAC	HE578137
**CXCL14**	TCCCTACAAGCTGAAGCCTAC	TGCTTATCCTTCCAGATCCG	HE578138
**IFNγ**			
**IFNγ1**	CAAACTGAAAGTCCACTATAAGATCTCCA	TCCTGAATTTTCCCCTTGACATATTT	AJ616215
**IFNγ2**	CAAACTGAAAGTCCACTATAAGATCTCCA	GGTCCAGCCTCTCCCTCAC	FM864345

Table S1. Continued.

Gene	Forward (5' to 3')	Reverse (5' to 3')	Acc. No.
**IL-17 family**			
**IL-17A/F1a**	CAAACGTACACTTTTTGATGGTGCTG	GGGACTCATCATAGGTGGTGTTGGT	KJ921977
**IL-17A/F1b**	AGGACCACAACACAGCAACAGA	CATGTAGGGATTGAGCCTTCAACC	KJ921978
**IL-17A/F2a**	CACCCTGGACCTGGAAAAGCAC	GGCCACAGACAGGAAGGAGG	AJ580842
**IL-17A/F2b**	CGCACCTTCCTCCACTCAAG	AGTCAATTCTTCTGGCTTTGTGGG	KJ921979
**IL-17A/F3**	GCAACGTTTCACTCTGAAGAGACAG	CTGGGGAAGTAGACAAAGGGCATC	KJ921980
**IL-17N**	ACCCACAGCACAGCAGAGACAGA	GATGTCATTGTGGCAAACGACTAAC	KJ921981
**IL-17C1**	CTGGCGGTACAGCATCGATA	GAGTTATATCCATAATCTTCGTATTCGGC	FM955455
**IL-17C2**	CTGGCGGTACAGCATCGATA	CAGAGTTATATGCATGATGTTGGGC	FM955456
**IL-17D**	GAAGAAATCCTCGAGCAGATGTTTG	GGGTCGTGGGAGATCCTGTATG	AJ580843
**γ-chain cytokines**			
**IL-2**	TGATGTAGAGGATAGTTGCATTGTTGC	GAAGTGTCCGTTGTGCTGTTCTC	AM422779
**IL-4/13A**	ACCACCACAAAGTGCAAGGAGTTCT	CACCTGGTCTTGGCTCTTCACAAC	FN820501
**IL-4/13B1**	GAGATTCATCTACTGCAGAGGATCATGA	GCAGTTGGAAGGGTGAAGCTTATTGTA	HG794522
**IL-4/13B2**	GAGACTCATCTATTGCGTATGATCATCG	TGCAGTTGGTTGGATGAAACTTATTGTA	HG794523
**IL-15**	TGGAATTGCTTCATAATATTGAGCTGCC	TGGTAGTTATCTGTGACCGACATGTCCTC	AJ628345
**IL-21**	AAAGTTATCAAAAACCTCAACAACCGAA	CCAGTCTACTGATGGCCTTTTGAAG	FM883702
**Anti-inflammatory cytokines**			
**IL-10A**	GGATTCTACACCACTTGAAGAGCCC	GTCGTTGTTGTTCTGTGTTCTGTTGT	AB118099
**IL-10B**	GGGATTCTAGACCACATCAAGAGTCC	GATGGGAGATTTAAAGTTGTGTGTTCC	FR691804
**TGFβ1A**	CTCACATTTTACTGATGTCACTTCCTGT	GGACAACTGCTCCACCTTGTG	OMY7836
**TGFβ1B**	CATGTCCATCCCCCAGAACT	GGACAACTGTTCCACCTTGTGTT	FN822750
**Other cytokines**			
**IL-20**	CAAGAACCTGAGGCAATGTCACTG	TCTCCTATAGCCTTTACTGCTGCCG	FN386780
**IL-22**	GAAGGAACACGGCTGTGCTATTAAAC	GATCTAGGCGTGCACACAGAAGTC	AM748538
**IL-34**	AGGCAGAAGACGTAACATGAAACACA	TCCACCCTCGCCCTCAGCTT	FN820429
**SOCS genes**			
**CISHa1**	CATTCTACCTTGATACCTCAGGCTGGT	CCTGCTGCACCTTCCTCCC	AM903340
**CISHa2**	TCTTCTACCTTGATACCTCAGGCTGGT	CCTTGCCCTTCTGTACCTTCCTTGT	HG003693
**CISHb1**	GAATGATGGTGAGAGAGGAGATTTGTGT	GACCCCCAGTACCAACCTGAGTT	FR873795
**CISHb2**	GATCATGGTGAGAGAGGAGGGTCA	GACCCCCAGTACCAACCTGAGTT	HG003694
**SOCS1A**	GCTAGGAGTGGAGGAGGCTCACA	AGAGGCAGGGGAAAGAGTGCTC	AM748721
**SOCS1B**	AGCTTCCTCATCAGGGACAGCA	GCCTATGGGATGACTCCACGTAGAA	KY387584
**SOCS2A1**	CAAAAACAAGACGCGTGGGAGAA	CGTCGGAATCTACAACCCGTGACA	AM748722
**SOCS2A2**	CCACTCGCAAATAGGACGATTCATTT	TCATCGGAGTCTACAACTCGCGACT	KY387585
**SOCS2B**	CTCACCTTCAGGAGTCAGGCTGGTA	ACATGCCAGTGGCGTGCTCTATAC	FR874096
**SOCS3A**	CACAGAGAAACCGTTAAAAGGACTATCC	AAGGGGCTGCTGCTCATGAC	AM748723
**SOCS3B1**	CACATGCACACTACCAACAGGTGCTGT	GGGGCATGTGTGTGCAGCA	KY387586
**SOCS3B2**	CACGCACACTACCAACAGGTGCTGT	GGGCGTGTGCGTGCAGTG	KY387587
**SOCS4**	CGTTGTCTCTCTAAAACTATTTTTAAAAGCACAGAG	AGGTGGAACGCGCTCCTTG	KY387588
**SOCS5A1**	GTGTTTATAAAAGACAAAAGGGTCTTGGTCTC	GACGTCGTCCCCCAGTAACCAT	KY387589
**SOCS5A2**	CTGGTTTAAAAAGACAAAAGGGGCTTG	TCCTCATTGTCTTTTCTGTCTGTGGTTC	KY387590
**SOCS5B1**	GCAAGGAATACTGTTTTTTTTAAGCCTCAAG	GACGTATGGCCTTTTTTCTTTCCTCTTC	AM903341
**SOCS5B2**	GAACAACGAAGTTTTTATGCCCCAAGT	CAGCTTGCTCATGGGACGTCTG	KY387591
**SOCS6A1**	GTTGAAAAACATACTGTAGAAAGGGATGACA	TCCTCATGATTGAAGTCGTTGCG	AM903342
**SOCS6A2**	GTTTTTAACAGAGGATGACAGGGATGACT	CTTCTCCTCGTGATCAAAATCGTTACA	KY387592
**SOCS6B1**	TGCAGATGTTCATCTAACGAAAGCAAAG	TTGGCAGGGGCGGCTACTCT	KY387593
**SOCS6B2**	GCAGATGGTAATCTAACGAAGGGATGAGT 70	CCCTCCTTCTTCCTTGCCCTTGT	KY387594

Table S1. Continued.

Gene	Forward (5' to 3')	Reverse (5' to 3')	Acc. No.
**SOCS genes**			
**SOCS7A1**	GGACCAGGACACAGGGAGCAAACT	GAAGGCAGGACCGAAGGCCAC	AM903343
**SOCS7A2**	CAGACTTGGACACAGGGAGCAAACA	GAAGGCAGGACCGAAGGCCAA	KY387595
**SOCS7B1**	CGCTGCCCAAACCACTGATCA	CCAGTTACGTCTGAGACTCCGCCTCTTT	KY387596
**SOCS7B2**	CGCTGCCCAAACCACTGATCA	CAGTTACGTCTGAGACTCGGCCACTTG	KY387597
**APPs**			
**SAA**	GGTGAAGCTGCTCAAGGTGCTAAAG	GCCATTACTGATGACTGTTGCTGC	AM422447
**SAP1**	GCTGTTATGGTGACCTTCAAGATCTCTC	GCGTTTGTACAACAACAAATCATTGTC	X99385
**SAP2**	GGTTGTTATGCTGAACATCAAGATCTCTC	CCACCCTTTGATTGCATACACAGATT	EZ763346
**AMPs**			
**CATH1**	ACCAGCTCCAAGTCAAGACTTTGAA	TGTCCGAATCTTCTGCTGCAA	AY594646
**CATH2**	ACATGGAGGCAGAAGTTCAGAAGA	GAGCCAAACCCAGGACGAGA	AY542963
**Hepcidin**	GCTGTTCCTTTCTCCGAGGTGC	GTGACAGCAGTTGCAGCACCA	CA369786
**β-defensin 1**	GGTTTTCCTATTGCTTAATGTTGTGG	GACACACAGTTAAGTCATGG	AM282655
**β-defensin 2**	GCTGACAGCAGTGCAAGCTGATGACAC	GCAAAGCACAGCATCTTAATCTGC	FM212656
**β-defensin 3**	GCTTGTGGAATACAAGAGTCATCTGC	GCATACATTCGGCCATGTACATCC	FM212657
**β-defensin 4**	GCAACTCTTCTAAAGAACAGT	CGTGGGCGACACAGCATACAAATCC	FM212658
**LEAP2A**	GGTTCCTGGTGTTTCTGGTGCT	AGTGGCCACCCCTGCAAAT	AY362186
**LEAP2B**	TGCACGGTCAAAACCACAGCA	AGGAATGAACTGCCCCACTGA	AY362187
**Complement genes**			
**Bf-1**	ACAATTGTATCGAAATGGCTGTTACTG	CCACCGGAGTCACCTTTGC	AF089861
**Bf-2**	GGCGAATTGTATTGAATTGGCTACTA	CCACCGGAGTCACCTTTGC	AF089860
**C1r**	CCTCCCTGAGTGCCACATAATTG	TTGTCTCTCCATGTTCTATCTGCTGC	AJ519930
**C1 inhibitor**	AACAGTTGGGTTGCAAAGCAGAC	GGAACATCGCCACCTGTGC	AJ519929
**C3-3**	TTGGCTGTGGATTGAGGAGAGTCT	GGGTTCAGACACACAGATTCCATG	U61753
**C3-4**	GGATGACCGATTCGAAAGTTTGG	GGGTTCAGACACACAGATTCCATG	AF271080
**C4**	ACAGACGACCTGGACAAGCTAAAGA	CATCCCCTGAACACAATGTTGAAAC	AJ544262
**C5**	GAGGAAGATCAGAGAGGGCATCAC	CTTTGACGTATTTGTCCACCTGTCC	AF349001
**C6**	CATCTCCATTTTCCAAGAGCAAAATG	GAGCACACCTTCCTGATGCACA	AJ622903
**C7-1**	CTTCCATACCAATGCCAGCCTTC	ATCCGACCAATCACAATCACTGTC	AJ566190
**C7-2**	AAATGCCTTATGAGTGCACGTCTTC	TTGTCCGTCACAAGTCTCCCA	AJ622902
**C8α**	GAGATGTATGGCAGCTCCAGTTTGA	CAAATATGGGCAGGGTCTCAAACTC	NM_001124624
**C8β**	GGCTGTTCTCAAAGGCACCA	GCCTCCGCTGCTAGGTGC	NM_001124607
**C8γ**	AGCACTACCACACGCCTTAACC	GGTGAGCTGTCCCTGTTTCTG	AJ622904
**C9**	GCGCTGGTGGATAAGGTGATAACTC	GAATGGGTTCTGGCTCACTGCT	NM_001124426
**CR1 like**	CCTACTCCCACATGCAAAGATGGTG	CCTATAACACAAACAAAACCAAGTCCACC	NM_001124404
**C3aR**	ATTTAAGAAAGGAAAGCTCAAAGATGG	GTCAGCTATGGAGAAGGGTATGGA	AJ616902
**C5aR**	CCATACGCCTGAAATCTAAGTCACCTC	ACCCAGCAGGAAGACCAAGCC	NM_001124467
**Arginase genes**			
**Arginase 1a**	CAGAGGTGGATCGCCTTGGAATA	GCAGACAGCATCCCTGTCTGACA	KX998965
**Arginase 1b**	AGGTGGATCGCCTTGGAATCG	GCAGACAGCAGCCCTGTCTGACT	KX998966
**Arginase 2a**	TCCAGAGAGTCATGGAAGTCACTTTCC	CCATCACTGACAACAACCCTGTGTT	KX998967
**Arginase 2b**	TCCAGAGAGTCATGGAAGTCTCTTTCG	CATCACCGACAACAACCCTGTGTT	KX998968
**TLR5**			
**TLR5S**	GCGCATCACTTCAGGGGGAT	GCATTTCACCACTTGCAGGTAGA	AB062504
**TLR5M**	GCGCTCATAACTTCAGGGGGAT	GCATTTCACCACCTGCAGGTATT	AB091105

Table S2. Fold change of transcript expression of immune-relevant genes in the spleen, liver, gills and skin of fish ip injected with YRF. The fold change was calculated as the average expression level of YRF injected fish divided by that of time matched controls (N=7). Asterisks indicate significant differences (One-way-ANOVA with Bonferroni correction) between YRF injected fish and controls, as follows; * = $p < 0.05$, ** = $p < 0.01$ and *** = $p < 0.001$.

**Tissue**	Spleen		Liver		Gills		Skin	
**Time**	6h	24h	6h	24h	6h	24h	6h	24h
**IL-1 Family**								
**IL-1β1**	138.1***	2.3	166.2***	10.1***	47.7***	1.9	468.9***	3.5
**IL-1β2**	3702.6***	4.5*	186.4***	2.7	259.7***	3.3	166.4***	3.1
**IL-1β3**	1.3	0.6	1.8	0.8	3.6***	0.7	4.1***	1.1
**nIL-1Fm**	5.7***	1.1	4.3***	2.0	2.1	1.0	2.6*	1.2
**IL-18**	2.7***	1.2	1.5	4.9***	1.2	1.2	1.2	1.5
**TNF family**								
**TNFα1**	12.8***	0.9	12.6***	1.0	10.4***	0.7	24.3***	1.0
**TNFα2**	24.4***	2.9	272.5***	13.8	20.8***	1.4	206.6****	3.1
**TNFα3**	1.6	0.4	73.7***	1.5	2.4	1.7	47.7*	0.5
**IL-6 family**								
**IL-6**	999.6***	6.2**	463.5***	7.8**	68.0***	3.4	240.1***	7.7***
**IL-11**	92.8***	9.7***	1766.1***	22.3***	34.0***	4.3*	112.8***	9.7***
**M17**	14.7***	6.0***	6.3***	2.2	7.3***	5.4***	4.3**	3.1
**CNTF**	0.5	0.9	0.8	0.6	1.0	0.6	1.0	0.6
**IL-12 family**								
**P19**	10.0***	2.4	1391.6****	64.2***	4.0	0.4	68.7***	1.4
**P35A1**	1.1	0.9	30.9***	2.4*	1.4	1.2	40.7***	3.9
**P35A2**	51.9***	2.1	68.3***	0.2	20.8***	1.6	270.7***	1.1
**P35B1**	2.4	0.3	9.0	0.1	1.6	0.8	13.9	0.9
**P28A**	2.3	0.9	ND	ND	13.4	1.5	7.5	0.4
**P28B**	5.9	2.0	ND	ND	ND	ND	ND	ND
**P40B1**	ND*	ND	ND*	ND	2.1	0.8	3.9	0.8
**P40B2**	3.1	2.4	2.0	4.4	1.6	0.8	2.0	1.3
**P40C**	1.2	0.5	2.4	1.3	2.1	0.7	1.5	0.8
**EBI3**	5.0**	0.3	1.3	0.6	1.2	1.0	0.7	0.7
**Chemokines**								
**CXCL_F1a**	22.2	103.0	1.5	1.0	0.6	0.7	1.6	0.8
**CXCL_F1b**	0.8	12.2	0.7	2.7	1.5	2.6	1.2	1.6
**CXCL_F1c**	1.1	0.4	0.8	1.2	1.1	0.2***	0.7	0.4
**CXCL_F2**	1.1	0.3	2.0	0.7	1.1	0.5	0.7	0.6
**CXCL_F4**	22.9***	8.0**	38.9***	32.3***	43.4***	10.7***	47.6***	18.9***
**CXCL_F5**	7.4***	2.2	6.5***	1.1	1.6	1.2	4.6***	1.9
**CXCL8**	344.1***	14.0***	82.9***	13.3***	11.2***	1.5	120.8***	3.0
**CXCL11_L1**	1.2	0.5	1.2	1.4	0.7	0.4	1.6	0.3
**CXCL12a**	0.9	0.4	1.8	0.4	0.8	1.6	0.9	1.5
**CXCL12b**	0.8	0.4	0.7	1.2	0.8	1.7	0.5	0.8
**CXCL13**	2.7	15.7***	0.7	99.7***	0.8	19.0***	0.5	12.6**
**CXCL14**	1.6	0.7	0.8	0.6	1.0	1.5	0.9	1.8
**IFNγ**								
**IFNγ1**	1.3	0.7	1.0	3.8	0.3	0.9	0.2	0.4
**IFNγ2**	1.0	1.9	1.3	1.3	0.4	0.8	0.5	0.5
**IL-17 family**								
**IL-17A/F1a**	364.5***	233.0***	17.7***	29.3***	0.5	0.3	0.5	0.9
**IL-17A/F1b**	ND	17.5	ND	ND	1.2	0.6	0.3	0.6
**IL-17A/F2a**	0.6	0.7	1.9	11.4***	0.4	0.5	0.2	0.4
**IL-17A/F2b**	0.8	1.1	2.0	2.2	0.9	1.0	0.5	0.9
**IL-17A/F3**	0.6	1.2	1.6	0.6	0.7	1.1	0.4	0.7
**IL-17N**	1.9	1.6	1.1	1.2	2.2	1.0	0.9	0.8
**IL-17C1**	1.9	4.1	4967.4***	718.4***	7.4***	0.5	21.0***	0.8
**IL-17C2**	3.6	4.1	130.5***	11.7***	3.4	0.6	7.8***	4.2
**IL-17D**	0.9	0.4	0.4	0.6	1.0	0.8	0.6	0.4

Table S2. Continued.

Tissue	Spleen		Liver		Gills		Skin	
**Time**	6h	24h	6h	24h	6h	24h	6h	24h
**γ-chain cytokines**								
**IL-2**	1.1	0.7	1.2	1.1	1.0	0.6	1.0	1.1
**IL-4/13A**	0.9	1.1	0.7	4.6**	0.9	1.5	1.1	0.8
**IL-4/13B1**	0.1	3.5	0.4	13.1***	0.3	2.2	0.7	0.9
**IL-4/13B2**	1.2	1.6	0.8	1.1	1.2	1.0	1.2	0.6
**IL-15**	1.0	0.6	2.7***	8.4***	2.4*	0.7	1.2	1.0
**IL-21**	1.6	0.9	1.7	1.4	1.5	0.8	0.9	0.9
**Anti-inflammatory cytokines**								
**IL-10A**	4.3	7.9	5.5	0.4	0.6	1.8	1.7	1.4
**IL-10B**	0.9	1.1	5.9	0.7	1.5	1.0	1.4	1.4
**TGFβ1A**	1.6	1.4	1.6	3.3***	1.1	1.4	0.9	1.2
**TGFβ1B**	1.7	1.3	22.8***	0.4	2.0	1.4	1.6	0.8
**Other cytokines**								
**IL-20**	6.5	1.2	2.0	2.1	1.0	1.8	1.0	1.1
**IL-22**	8.9**	3.3	112.1***	12.5***	6.5*	0.6	68.6***	1.6
**IL-34**	2.9***	1.0	18.7***	0.7	1.7	1.1	3.4***	1.5
**SOCS genes**								
**CISHa1**	1.4	3.3	8.0***	0.2***	2.4	0.8	4.4**	1.0
**CISHa2**	5.4*	2.2	19.6***	0.2**	6.6**	1.0	8.3***	0.9
**CISHb1**	1.9	2.5	1.4	0.8	1.2	0.6	1.2	0.8
**CISHb2**	2.8	2.1	0.9	1.0	1.4	1.0	1.8	1.0
**SOCS1A**	3.6**	1.7	22.2***	1.4	3.0*	1.2	3.1**	1.3
**SOCS1B**	1.2	1.4	0.5	2.8	0.8	1.2	0.9	1.3
**SOCS2A1**	2.4	3.7	1.1	0.3	1.3	1.8	1.0	1.3
**SOCS2A2**	0.6	0.5	3.3	0.2	1.4	1.2	0.7	0.8
**SOCS2B**	1.0	0.2	2.5	0.4	1.2	0.3	1.1	0.3
**SOCS3A**	11.0***	6.0**	38.6***	3.8	5.7***	2.4	3.6*	2.6
**SOCS3B1**	1.5	1.5	1.7	3.8	1.0	2.5	2.2	3.4
**SOCS3B2**	7.0*	6.2	2.6	16.2**	2.5	4.7	2.5	6.9
**SOCS4**	1.3	0.3	0.3	0.5	1.8	1.7	0.8	1.3
**SOCS5A1**	1.2	1.5	0.1	2.3	1.2	1.8	0.8	2.4
**SOCS5A2**	0.6	1.3	0.1	0.9	0.9	1.6	1.0	1.6
**SOCS5B1**	1.0	1.0	0.4	1.1	1.0	1.4	1.4	1.3
**SOCS5B2**	1.4	1.1	0.7	1.4	2.8	1.7	2.8	2.4
**SOCS6A1**	0.7	1.0	0.4*	1.3	0.7	1.3	0.6	1.1
**SOCS6A2**	1.0	1.1	0.4***	1.3	1.2	1.0	0.6	1.0
**SOCS6B1**	1.0	1.0	0.4	0.8	1.2	1.4	0.5	0.9
**SOCS6B2**	0.8	1.1	0.3**	1.5	1.0	1.3	0.9	1.1
**SOCS7A1**	1.4	1.0	0.6	1.2	1.7	1.3	1.6	1.3
**SOCS7A2**	0.9	0.6	1.5	0.9	1.6	1.0	0.9	1.2
**SOCS7B1**	3.0	2.0	1.5	1.6	1.8	2.5	0.7	1.4
**SOCS7B2**	1.6	1.4	2.2***	1.7*	1.1	1.4	0.7	1.2
**APPs**								
**SAA**	31,1***	15.9***	225.8***	5450.6***	2.8	26.2***	8.7**	111.8****
**SAP1**	3.9*	3.2	1.4	0.6	0.4	3.5	0.6	0.8
**SAP2**	1.8	1.8	0.9	1.7	1.1	0.9	0.9	2.1

Table S2. Continued.

Tissue	Spleen		Liver		Gills		Skin	
**Time**	6h	24h	6h	24h	6h	24h	6h	24h
**AMPs**								
**CATH1**	39.0***	5.7	5.2	45.0***	4.3	10.4	29.0***	60.6***
**CATH2**	69.0***	26.6****	745.0***	14687.1****	19.9***	137.5***	10.7***	128.5***
**Hepcidin**	278.6***	75.4***	11.9***	235.2***	65.4***	309.5***	66.0***	1891.5***
**β-defensin 1**	ND	0.0	ND	ND	1.8	0.3	0.9	1.2
**β-defensin 2**	3.1	5.4	1.5	0.8	0.8	0.7	0.3	1.2
**β-defensin 3**	4.4	1.3	0.9	1.8	7.6	3.3	3.0	1.3
**β-defensin 4**	0.9	3.1**	0.5	2.7*	0.9	1.5	0.7	1.1
**LEAP2A**	0.6	1.3	2.6	0.04***	0.5	0.8	1.3	1.1
**LEAP2B**	0.8	0.2	1.2	0.05***	1.3	0.6	0.7	0.6
**Complement genes**								
**Bf-1**	13.6***	4.3	1.6	1.8	0.8	1.3	0.7	0.8
**Bf-2**	1.9	1.9	1.7	1.4	1.4	0.9	0.2	0.5
**C1r**	4.0**	1.0	1.1	0.7	1.4	1.3	0.8	0.8
**C1 inhibitor**	2.8	1.1	1.0	0.7	0.8	1.2	0.8	1.3
**C4**	0.8	0.9	1.2	0.9	1.2	1.9	0.6	3.2
**C5**	7.8	1.4	1.3	1.3	1.1	0.7	0.2	1.2
**C6**	1.3	1.3	1.1	1.4	1.5	2.2	1.7	6.6***
**C7-1**	23.1***	19.5***	25.5***	23.2***	11.1***	12.4***	8.7***	12.5***
**C7-2**	1.2	0.7	0.9	0.8	0.9	1.2	1.3	0.7
**C8α**	5.4	1.5	0.9	0.8	1.3	0.3	0.6	0.7
**C8β**	1.9	1.0	1.3	0.6	1.2	0.9	0.7	0.8
**C8γ**	0.9	0.5	1.2	0.6	1.2	1.4	1.1	0.8
**C9**	1.0	1.0	1.5	1.8	0.6	0.9	0.6	0.5
**C3-3**	4.0	3.7*	1.0	1.3	0.6	2.0	0.4	1.4
**C3-4**	6.2*	2.0	1.1	0.5	1.8	0.9	0.9	0.6
**C3aR**	1.8	1.7	1.5	1.5	2.5	1.1	1.9	1.6
**C5aR**	4.6**	2.5	2.2	7.0***	2.9	5.3**	3.6	2.9
**CR1 like**	3.8***	3.1***	1.7	4.3***	2.7***	1.5	2.6***	2.2**
**Arginase genes**								
**Arginase 1a**	31.2***	11.5***	0.8	0.4	1.2	0.9	3.3	2.0
**Arginase 1b**	2.2	1.3	0.8	0.7	0.5	1.0	3.2	1.0
**Arginase 2a**	8.2***	3.1**	6.0***	6.5***	1.5	1.5	4.5***	3.5
**Arginase 2b**	16.3***	5.0**	15.8***	18.9***	3.6	2.6	5.8	3.6
**TLR5**								
**TLR5S**	25.2***	1.0	254.5****	23.4***	59.4***		274.4****	476.6***
**TLR5M**	1.0	0.2	2.6	0.0	0.5	0.9	0.7	1.6

Note

ND, not determined due to low expression levels.

ND*, detectable in YRF injected fish but not detectable in time-matched controls.
